# Prognostic value of estimated pulse wave velocity for all-cause and cardiovascular mortality in individuals with cardiovascular–kidney–metabolic (CKM) syndrome: analyses of NHANES 2007–2018

**DOI:** 10.1186/s13098-026-02174-4

**Published:** 2026-05-14

**Authors:** Zhongxing Zhou, Ruming Shen, Jiaxing Ke, Shuaijie Chen, Longqing Chen, Hailin Zhang, Xiaoyan Lin, Jinxiu Lin, Dajun Chai

**Affiliations:** 1 Department of Cardiology, the First Affiliated Hospital; Department of Cardiology, National Regional Medical Center, Binhai Campus of the First Affiliated Hospital, Fujian Medical University, 20 Chazhong Road, Fuzhou, 350005 China; 2 The Higher Educational Key Laboratory for Cardiovascular Disease of Fujian Province, Clinical Research Center for Metabolic Heart Disease of Fujian Province, The First Affiliated Hospital, Fujian Medical University, Fuzhou, 350005 China; 3 Department of Ultrasound, the First Affiliated Hospital of Fujian Medical University, Fuzhou, 350005 China

**Keywords:** Estimated pulse wave velocity (ePWV), Cardiovascular-kidney-metabolic (CKM), Prognosis, Machine learning

## Abstract

**Background:**

The impact of estimated pulse wave velocity (ePWV) on the prognosis of cardiovascular-kidney-metabolic (CKM) syndrome has not been explored. This study investigated the association between ePWV and mortality and its predictive performance in individuals with CKM syndrome.

**Methods:**

This population-based prospective study of 9,416 American participants used ordinal logistic regression to assess the association between ePWV and CKM syndrome severity. Cox regression was applied to evaluate the relationship between ePWV and all-cause and cardiovascular mortality at different CKM stages, as well as their interaction effects on mortality. To evaluate the predictive performance, the area under the curve (AUC) was calculated, and the predictive capabilities of the combined model incorporating both CKM syndrome and ePWV were compared with CKM syndrome alone. Furthermore, various machine learning models were developed for prediction.

**Results:**

ePWV was found to be significantly associated with the severity of CKM syndrome, with a common odds ratio (cOR) of 1.73 [95% confidence interval (CI): 1.68–1.77] per 1 m/s increase in ePWV. Moreover, ePWV demonstrated a significant association with both all-cause and cardiovascular mortality. Specifically, the hazard ratios (HR) per 1 m/s increase in ePWV were: 1.60 [95% CI: 1.51–1.69] for all-cause mortality in participants with early-stage CKM, 1.29 [95% CI: 1.23–1.35] for all-cause mortality in participants with advanced-stage CKM, 1.84 [95% CI: 1.63–2.08] for cardiovascular mortality in participants with early-stage CKM, and 1.23 [95% CI: 1.13–1.34] for cardiovascular mortality in participants with advanced-stage CKM. A notable interaction effect between ePWV and CKM syndrome was observed for both all-cause and cardiovascular mortality (P for interaction < 0.001). ePWV significantly enhanced the predictive performance of CKM syndrome for all-cause and cardiovascular mortality [the net reclassification improvement (NRI) across different time points ranged from 0.25 to 0.382 for all-cause mortality and from 0.005 to 0.431 for cardiovascular mortality]. Furthermore, the combination of ePWV and CKM syndrome demonstrated strong predictive power in most models (time-dependent AUC > 0.7).

**Conclusion:**

ePWV serves as a critical indicator for both the severity and the mortality risk among individuals with CKM syndrome. ePWV significantly enhanced the accuracy of mortality risk prediction of CKM syndrome.

**Graphical Abstract:**

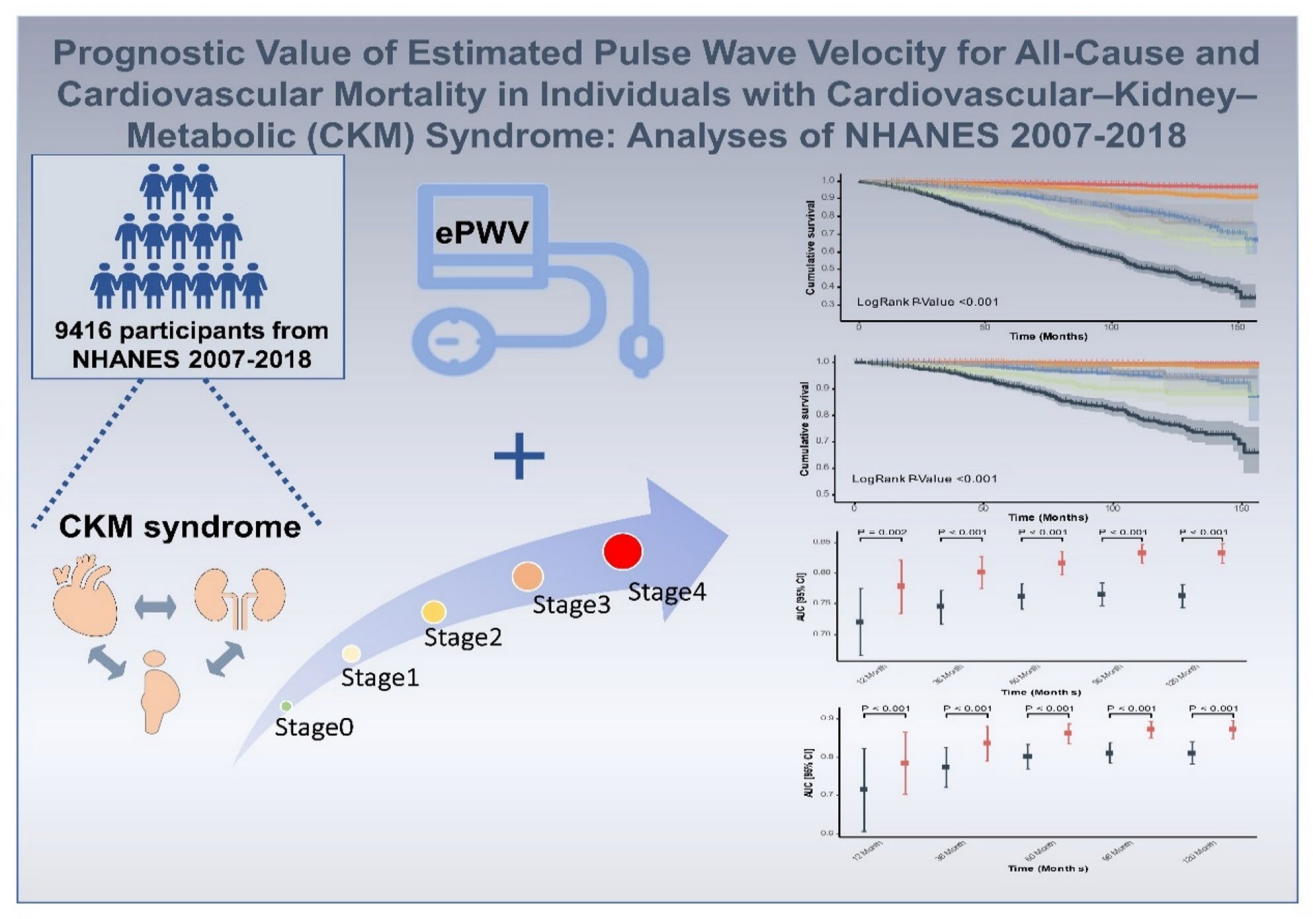

**Supplementary Information:**

The online version contains supplementary material available at 10.1186/s13098-026-02174-4.

## Research Insights

What is currently known about this topic?

The high prevalence of suboptimal CKM syndrome represents a significant public health concern.

What is the key research question?

To elucidate the role of ePWV in prognostic value for mortality outcomes in the CKM population.

What is new?

The integration of ePWV with CKM syndrome demonstrates a significant improvement in the predictive performance of CKM syndrome for mortality outcomes.

How might this study influence clinical practice?

Incorporating ePWV into CKM syndrome risk stratification may contribute to early identification of high-risk individuals and enable timely, targeted interventions.

**Supplementary Information:**

The online version contains supplementary material available at 10.1186/s13098-026-02174-4.

## Background

Mounting evidence underscores the significant interplay among cardiovascular disease (CVD), chronic kidney disease (CKD), and metabolic abnormalities. Metabolic abnormalities are both bidirectionally and causally linked to CVD and CKD, with CKD acting as a pivotal mediator between metabolic abnormalities and CVD [1, 2]. Poor cardiovascular-kidney-metabolic (CKM) health is a major risk factor for increased morbidity, multi-organ disease, and premature mortality [1]. To improve the integrated management of CVD, CKD, and metabolic diseases, the American Heart Association (AHA) has proposed the concept of cardiovascular-kidney-metabolic (CKM) syndrome [3, 4]. Studies have shown that nearly 90% of U.S. adults meet the diagnostic criteria for CKM syndrome (stage 1 or higher), while 15% fulfill the criteria for advanced stages of the syndrome [5]. Therefore, there is an urgent need to improve the management of individuals with CKM syndrome and to actively identify simple and practical risk biomarkers.

CKM syndrome provides a comprehensive framework for evaluating cardiovascular, renal, and metabolic diseases [3, 4]. However, it does not currently incorporate measures of peripheral artery abnormalities, which are bidirectionally associated with CVD, CKD, and metabolic abnormalities. Metabolic syndrome has been recognized as a condition linked to increased risk of peripheral artery abnormalities [6–8]. Peripheral artery abnormalities impair organ perfusion and exacerbating injury to the heart and kidneys [9–11]. Therefore, indicators of peripheral artery abnormalities may serve as important risk markers for both the severity of CKM syndrome and mortality risk among individuals with CKM syndrome. Incorporating such measures could potentially enhance the mortality predictive performance of the CKM syndrome.

Carotid-femoral pulse wave velocity (cfPWV), ankle–brachial index (ABI), and intima–media thickness (IMT) are commonly used indicators of peripheral vascular abnormalities [12]. However, their acquisition requires precise pressure sensing devices and ultrasonic transducers, which substantially limits the large-scale data collection. Estimated pulse wave velocity (ePWV) is calculated using two easily accessible parameters, blood pressure and age, through a simple algorithm, and has been demonstrated to have similar values to measured cfPWV, the gold standard for the assessment of arterial stiffness [13, 14]. Multiple studies have demonstrated that ePWV predicts cardiovascular events, cerebrovascular events, and all-cause mortality independently of traditional risk factors [15–17]. In accordance with the principle that risk prediction metrics should be readily accessible, our study focuses on ePWV.

We acknowledge that previous studies have established the association between ePWV and mortality in patients with single disease such as hypertension, diabetes, CKD, and CVD [18–20]. However, individuals with coexisting CVD and CKD demonstrate a tenfold higher mortality risk than those with CVD alone [21, 22]. This highlights that multimorbidity is associated with a significantly higher risk of mortality compared to the presence of a single disease. However, research focusing on the role of ePWV in predicting outcomes among individuals with CKM multimorbidity remains scarce. Therefore, it is crucial to examine the relationship between ePWV and mortality within the framework of CKM syndrome.

In this study, we first investigated the association between ePWV and the severity of CKM syndrome. We then examined the relationship between ePWV and mortality risk among individuals with different severities of CKM syndrome. Furthermore, we explored the interactive effects of ePWV and CKM syndrome on mortality risk in the community population. Finally, using Cox regression and machine learning approaches, we assessed whether incorporating ePWV could improve the predictive performance of CKM syndrome for mortality in the general population.

## Methods

### Population

This population-based prospective study was conducted on 9416 participants aged 20 years or older from the National Health and Nutrition Examination Survey (NHANES) 2007–2018. Participants with incomplete data on the variables necessary for defining CKM syndrome and ePWV were excluded from the analysis. Detailed inclusion and exclusion criteria can be found in Supplementary Figure S1. The NHANES program received approval from the National Center for Health Statistics (NCHS) Ethics Review Board, and informed consent was acquired from all participants. Given the deidentified and anonymous characteristics of the NHANES data, no further ethical approval or informed consent was necessary for conducting secondary analyses.

### Estimated pulse wave velocity measurement and calculation

Blood pressure (BP) measurements were carried out on participants who did not have any of the following conditions: gauze dressings, edema, casts, paralysis, rashes, atrophied arms, arterial-venous shunts, tubes, open sores or wounds, or a history of radical mastectomy. Prior to initiating BP measurements, the circumference of the upper arm was assessed to determine the appropriate cuff size. Participants were then instructed to remain seated in a quiet and relaxed environment for a duration of five minutes. Following this, three consecutive BP readings were recorded, including systolic blood pressure (SBP) and diastolic blood pressure (DBP). In cases where an interruption or incomplete recording occurred during any BP measurement, a fourth attempt was allowed [23].

The average BP, encompassing both SBP and DBP, was calculated as follows: the diastolic reading of zero was excluded from the calculation of the average DBP; if all diastolic readings were zero, the average would consequently be zero; if only a single BP reading was recorded, that reading would represent the average; in cases where multiple BP readings were obtained, the first reading was always excluded from the calculation of the average.

The equation for calculating the ePWV was established by Greve et al. [14] in the Reference Values for Arterial Stiffness Collaboration [24]. The ePWV is computed using the individual’s age and mean blood pressure (MBP) according to the following formula:

$$\begin{aligned} ePWV&=9.587-0.402\times\:age+4.560\times\:{10}^{-3}\\& \quad \times\:{age}^{2}-2.621\times\:{10}^{-5}\\& \quad \times\:{age}^{2}\times\:MBP+3.176\times\:{10}^{-3}\\& \quad \times\:age\times\:MBP-1.832\times\:{10}^{-2}\times\:MBP \end{aligned}$$

MBP was calculated as DBP + 0.4 (SBP-DBP).

### Cardiovascular-kidney-metabolic syndrome

The stages of CKM syndrome were delineated based on the American Heart Association’s Presidential Advisory Statement on CKM Syndrome [1]. Stage 0 is characterized by the absence of any risk factors associated with CKM syndrome. Stage 1 is identified by the presence of excess or dysfunctional adiposity. Stage 2 encompasses individuals exhibiting metabolic risk factors along with CKD. Stage 3 is characterized by the presence of subclinical CVD, while Stage 4 is defined by the presence of clinical CVD. CKD stages were classified according to the Kidney Disease Improving Global Outcomes (KDIGO) guidelines, which are based on estimated glomerular filtration rate (eGFR) and albuminuria levels [25]. The eGFR was determined using the Chronic Kidney Disease Epidemiology Collaboration (CKD-EPI) creatinine Eq. 2021 [25]. Subclinical CVD is defined as a 10-year CVD risk of 20% or greater, as calculated by the PREVENT (Predict Risk of cardiovascular disease EVENTS) base model [3, 26]. Detailed definition of CKM stage can be found in Supplementary Methods.

### Mortality outcomes

Mortality data were obtained from the National Death Index (NDI), with cardiovascular mortality classified according to the International Classification of Diseases, Tenth Revision (ICD-10; codes I00-I09, I11, I13, I20-I51, I60-I69). The follow-up period was defined as the number of months from the Mobile Examination Center interview until all-cause mortality, cardiovascular mortality, or the end of the mortality follow-up period on December 31, 2019.

### Other variables

Other variables include socioeconomic factors (age, gender, ethnicity, income level, education, housing instability, regular health-care access, and food security), life style (sleep problem, physical activity, healthy eating index score, and smoking), depression, and medicine usage (anti-hypertension and cardiovascular improvement). Detailed definition can be found in Supplementary Methods.

### Statistics

Before the data analysis, we used the random forest method to impute the missing values (the pattern of missing data was presented in Supplementary Figure S2) [27]. Continuous variables were expressed as mean with standard deviation (SD) or median with interquartile range (IQR), depending on the distribution of the data. Categorical variables were reported as counts and percentages. Wilcoxon rank sum test and Pearson’s Chi-squared test were used to compare the group differences for continuous variables and categorical variables, respectively.

To investigate the association between ePWV and CKM stages, ordinal logistic regression models were developed. ePWV was treated as a continuous variable and categorized based on terctiles. Model 1 was unadjusted, while Model 2 was adjusted for gender, ethnicity, income, education, housing instability, regular health-care access, and food security. Model 3 included further adjustments for sleep problems, physical activity, healthy eating index score, smoking, depression, and antihypertensive medication. Due to the presence of multicollinearity between age and ePWV (variance inflation factor > 4), age was not included as a covariate in the models. To explore the non-linear associations, ordinal logistic regression models using restricted cubic splines (RCS) in Model 3 were employed. In cases where non-linear relationships were identified (P value for non-linear < 0.05), two-piecewise linear regression analyses were conducted using the segmented R package (Version 2.1-3) to determine the cutoff values [28, 29]. Subgroup analyses were conducted based on socioeconomic factors (age, gender, and ethnicity) and variables utilized to define CKM syndrome, such as hypertension, BMI, waist circumference, metabolic syndrome, glucose status, and CKD. The selection of these CKM-defining variables was informed by previous research indicating their association with ePWV [30–32].

Cox proportional hazards regression models were developed to examine the associations between ePWV and both all-cause mortality and cardiovascular mortality in populations with early CKM Syndrome (CKM stages 0–2) and advanced CKM syndrome (CKM stages 3–4). Model 1 was unadjusted, while Model 2 was adjusted for gender, ethnicity, income, education, housing instability, access to regular healthcare, and food security. Model 3 included further adjustments for sleep problems, physical activity, healthy eating index scores, smoking, and depression. Schoenfeld residuals were employed to test the proportional hazards assumption and identified that the use of medications with established cardiovascular benefits did not meet the proportional hazards assumption. Consequently, this variable was excluded from the model and instead analyzed as stratifying variables in subgroup analyses. RCS with a fully adjusted Cox regression model was utilized to explore potential non-linear associations. Subgroup analyses were conducted based on age, gender, hypertension, BMI, waist circumference, metabolic syndrome, glucose status, CKD, and the use of cardiovascular improvement medications as these factors influence ePWV, CKM stage, and mortality [30–32].

To explore the joint association between CKM stage and ePWV with all-cause and cardiovascular mortality, the interaction effect of CKM stages (early CKM syndrome and advanced CKM syndrome) and ePWV (continuous) was firstly estimated. Then a new variable based on CKM stages (early CKM syndrome and advanced CKM syndrome) and tertiles of ePWV was defined. Kaplan–Meier methodology was utilized to construct survival curves, while Cox regression models were employed to examine the associations between the joint effects of CKM stage and ePWV on all-cause and cardiovascular mortality.

Several sensitivity analyses were conducted. First, complete-case analyses were performed on participants without missing data to mitigate bias resulting from data imputation. Second, competing risk models were developed to estimate the association between ePWV and cardiovascular mortality in participants with early CKM syndrome and advanced CKM syndrome, as well as the joint association between CKM stage and ePWV with cardiovascular mortality, considering other causes of mortality as competing events.

We used the *timeROC* (version 0.4) R package to construct time-dependent ROC curves and calculate the area under curves (AUC) for mortality at different follow-up intervals for both the combined model (CKM syndrome with ePWV) and CKM syndrome alone [33]. Furthermore, the *survIDINRI* (version 1.1-2) R package was utilized to compute the net reclassification improvement (NRI) and integrated discrimination improvement (IDI) values for the combined model relative to CKM syndrome alone, with the number of iterations for perturbation-resampling set to 200 [34]. 

To ensure national representativeness, a complex sample analysis was conducted utilizing fasting sample weights (wtsaf2 year), following the principle of the “*highest proportion of missing values*”. Weighted ordinal logistic regression models were utilized to investigate the relationship between ePWV and CKM stages and weighted Cox proportional hazards regression models were employed to assess the association between ePWV and mortality outcomes among patients with varying CKM stages. All models were adjusted for the previously specified covariates. The *svykm* function from the *survey* package (version 4.4-2) was utilized to construct survival curves. Additionally, weighted Cox regression models were employed to examine the associations of joint effects of CKM stage and ePWV with both all-cause and cardiovascular mortality. The *timeROC* (version 0.4) R package were used to construct time-dependent ROC curves and calculate the AUC in national representative population [33].

Furthermore, the combined predictive performance of ePWV and variables used to define CKM syndrome was evaluated using the time-dependent AUC across various machine learning models, including Random Survival Forest (RSF), Gradient Boosting Machine (GBM), Cox model boosting (Coxboost), Survival Support Vector Machine (Survivalsvm), eXtreme Gradient Boosting (XGBoost), Supervised Principal Components (SuperPC), and Partial Least Squares Regression for Cox models (PLSRcox). Hyperparameter tuning was performed using 5-fold cross-validation combined with grid search, with hyperparameter search ranges detailed in Supplementary Table S1. For each model, confidence intervals of the AUC evaluation metric (from time-dependent ROC curves) were computed via 1000 bootstrap samples in both the training and validation sets. For all-cause mortality, model training was conducted on the NHANES participants and validated on the China Health and Retirement Longitudinal Study (CHARLS) dataset (see Supplementary Methods). In the case of cardiovascular mortality, since CHARLS did not provide the necessary data, the NHANES cohort was randomly partitioned, with 70% assigned to the training set and 30% to the validation set.

All statistical analyses were performed in R (version 4.3.2). *P* < 0.05 was considered significant (2-tailed).

## Results

### Baseline characteristics

Table 1 presents the baseline characteristics of the 9,416 participants included in this study. Among these, the largest group was found in CKM stage 2, consisting of 5,501 individuals (58.42%), while the smallest group was in CKM stage 0, with 395 individuals (4.19%). Compared to the population with low ePWV, those with elevated ePWV were older, more likely to be female, and exhibited higher values in BMI, waist circumference, and triglyceride levels. Additionally, this group demonstrated an increased likelihood of hypertension, diabetes, metabolic syndrome, and elevated risk of CKD. Supplementary Table S2 presents similar baseline characteristics of participants in complete-case analysis.

### Association between ePWV and CKM stages

Table 2 presents the findings regarding the association between ePWV and CKM stages. In the fully adjusted model (Model 3), the common odds ratio (cOR) for each 1 m/s increase in ePWV was 1.73 [95% confidence interval (CI), 1.68–1.77]. When comparing participants with the lowest ePWV, the cOR for those with the highest ePWV was 11.57 [95% CI, 10.11–13.27]. RCS indicated a non-linear relationship between ePWV and CKM stages, with a statistically significant non-linearity (P for non-linearity < 0.001, Supplementary Figure S3). Two piecewise linear regression analyses identified a cutoff value of 7.28 m/s (P value for likelihood ratio test < 0.001). For ePWV values less than 7.28 m/s, the cOR for each 1 m/s increase in ePWV was 2.63 [95% CI, 2.21–3.14], whereas for ePWV values equal to or greater than 7.28 m/s, the cOR was 1.65 [95% CI, 1.60–1.71]. Subgroup analyses demonstrated that the association between ePWV and CKM stages was consistent across different populations (Supplementary Figure S4). Additionally, the complete-case analysis indicated that data imputation did not have a significant impact on this association (Supplementary Table S3).

**Table 1:** Baseline Characteristics based on tertiles of estimated pulse wave velocity

ePWV, m/s					
Characteristic	Overall N = 9,416^1^	Q1, <7.79 N = 3,140^1^	Q2, 7.79-9.83 N = 3,138^1^	Q3, >9.83 N = 3,138^1^	p-value^2^
Age, year	56.58 (14.05)	42.05 (7.58)	56.36 (7.24)	71.34 (7.31)	<0.001
Gender					<0.001
Female	4,319 (45.87%)	1,276 (40.64%)	1,508 (48.06%)	1,535 (48.92%)	
Male	5,097 (54.13%)	1,864 (59.36%)	1,630 (51.94%)	1,603 (51.08%)	
Ethnicity					<0.001
Other Hispanic	1,118 (11.87%)	404 (12.87%)	419 (13.35%)	295 (9.40%)	
Mexican American	1,436 (15.25%)	597 (19.01%)	488 (15.55%)	351 (11.19%)	
Non-Hispanic Asian	720 (7.65%)	251 (7.99%)	256 (8.16%)	213 (6.79%)	
Non-Hispanic Black	1,868 (19.84%)	524 (16.69%)	717 (22.85%)	627 (19.98%)	
Non-Hispanic White	4,274 (45.39%)	1,364 (43.44%)	1,258 (40.09%)	1,652 (52.64%)	
Income					<0.001
Low	3,051 (32.40%)	1,050 (33.44%)	1,119 (35.66%)	882 (28.11%)	
Median	3,567 (37.88%)	1,095 (34.87%)	1,129 (35.98%)	1,343 (42.80%)	
High	2,798 (29.72%)	995 (31.69%)	890 (28.36%)	913 (29.09%)	
Education					<0.001
Less than high school	2,490 (26.44%)	755 (24.04%)	778 (24.79%)	957 (30.50%)	
High School graduate or higher	6,926 (73.56%)	2,385 (75.96%)	2,360 (75.21%)	2,181 (69.50%)	
Housing Instability					<0.001
Own home	2,978 (31.63%)	1,272 (40.51%)	957 (30.50%)	749 (23.87%)	
Rent home or other arrangement	6,438 (68.37%)	1,868 (59.49%)	2,181 (69.50%)	2,389 (76.13%)	
Regular health-care access					<0.001
At least one regular health-care facility	1,286 (13.66%)	666 (21.21%)	408 (13.00%)	212 (6.76%)	
None or emergency room	8,130 (86.34%)	2,474 (78.79%)	2,730 (87.00%)	2,926 (93.24%)	
Food security					<0.001
Full security	7,855 (83.42%)	2,510 (79.94%)	2,571 (81.93%)	2,774 (88.40%)	
Marginal Security	714 (7.58%)	291 (9.27%)	258 (8.22%)	165 (5.26%)	
Low Security	577 (6.13%)	225 (7.17%)	205 (6.53%)	147 (4.68%)	
Very low Security	270 (2.87%)	114 (3.63%)	104 (3.31%)	52 (1.66%)	
Sleep Problem					0.4
No	4,843 (51.43%)	1,644 (52.36%)	1,598 (50.92%)	1,601 (51.02%)	
Yes	4,573 (48.57%)	1,496 (47.64%)	1,540 (49.08%)	1,537 (48.98%)	
Regular physical Activity					<0.001
Yes	7,718 (81.97%)	2,670 (85.03%)	2,580 (82.22%)	2,468 (78.65%)	
No	1,698 (18.03%)	470 (14.97%)	558 (17.78%)	670 (21.35%)	
HEI Score	54.69 (13.53)	52.89 (13.41)	54.47 (13.36)	56.73 (13.55)	<0.001
Smoke					<0.001
Never	4,952 (52.59%)	1,771 (56.40%)	1,582 (50.41%)	1,599 (50.96%)	
Former	2,690 (28.57%)	606 (19.30%)	875 (27.88%)	1,209 (38.53%)	
Current	1,774 (18.84%)	763 (24.30%)	681 (21.70%)	330 (10.52%)	
BMI, kg/m^2^	28.40 (25.00,32.70)	28.00 (24.52, 32.23)	29.15 (25.70, 33.95)	28.04 (24.80, 31.81)	<0.001
Waist Circumference, cm	101.34 (15.32)	98.40 (15.52)	103.42 (15.63)	102.20 (14.33)	<0.001
Triglycerides, mg/dL	107.00 (75.00, 156.00)	102.00 (71.00, 155.00)	112.00 (78.00, 162.00)	107.00 (77.00, 151.00)	<0.001
HDL, mg/dL	53.71 (16.25)	51.80 (15.11)	53.39 (16.35)	55.94 (16.96)	<0.001
Hypertension					<0.001
No	3,510 (37.28%)	2,150 (68.47%)	933 (29.73%)	427 (13.61%)	
Yes	5,906 (62.72%)	990 (31.53%)	2,205 (70.27%)	2,711 (86.39%)	
Pre-Diabetes					0.4
No	2,705 (51.47%)	923 (52.68%)	883 (50.40%)	899 (51.34%)	
Yes	2,550 (48.53%)	829 (47.32%)	869 (49.60%)	852 (48.66%)	
Diabetes					<0.001
No	6,925 (73.55%)	2,795 (89.01%)	2,221 (70.78%)	1,909 (60.83%)	
Yes	2,491 (26.45%)	345 (10.99%)	917 (29.22%)	1,229 (39.17%)	
Chronic Kidney Disease					<0.001
Low risk	7,554 (80.23%)	2,922 (93.06%)	2,684 (85.53%)	1,948 (62.08%)	
Moderate risk	1,247 (13.24%)	173 (5.51%)	343 (10.93%)	731 (23.30%)	
High risk	375 (3.98%)	37 (1.18%)	73 (2.33%)	265 (8.44%)	
Very high risk	240 (2.55%)	8 (0.25%)	38 (1.21%)	194 (6.18%)	
Metabolic Syndrome					<0.001
No	5,400 (57.35%)	2,171 (69.14%)	1,620 (51.63%)	1,609 (51.27%)	
Yes	4,016 (42.65%)	969 (30.86%)	1,518 (48.37%)	1,529 (48.73%)	
Depression					0.008
No	8,611 (91.45%)	2,868 (91.34%)	2,837 (90.41%)	2,906 (92.61%)	
Yes	805 (8.55%)	272 (8.66%)	301 (9.59%)	232 (7.39%)	
Anti-hypertension Drug					<0.001
No	5,531 (58.74%)	2,682 (85.41%)	1,797 (57.27%)	1,052 (33.52%)	
Yes	3,885 (41.26%)	458 (14.59%)	1,341 (42.73%)	2,086 (66.48%)	
Medicine Usage					<0.001
No	6,263 (66.51%)	2,785 (88.69%)	2,037 (64.91%)	1,441 (45.92%)	
Yes	3,153 (33.49%)	355 (11.31%)	1,101 (35.09%)	1,697 (54.08%)	
CKM Stage					<0.001
Stage 0	395 (4.19%)	306 (9.75%)	73 (2.33%)	16 (0.51%)	
Stage 1	1,476 (15.68%)	988 (31.46%)	380 (12.11%)	108 (3.44%)	
Stage 2	5,501 (58.42%)	1,708 (54.39%)	2,264 (72.15%)	1,529 (48.73%)	
Stage 3	723 (7.68%)	7 (0.22%)	36 (1.15%)	680 (21.67%)	
Stage 4	1,321 (14.03%)	131 (4.17%)	385 (12.27%)	805 (25.65%)	

**Table 2:** Results for the association between ePWV and cardiovascular-kidney-metabolic syndrome stages

Estimated pulse wave velocity	Model 1	Model 2	Model 3
**Continuous**			
Per 1 m/s increase	1.83[1.79,1.87]	1.86[1.82,1.91]	1.73[1.68,1.77]
P Values	< 0.001	< 0.001	< 0.001
**Tertiles of ePWV**,** m/s**			
Q1, < 7.79	Ref	Ref	Ref
P Values	-	-	-
Q2, 7.79 ~ 9.83	3.97[3.57,4.43]	4.3[3.85,4.8]	3.13[2.79,3.5]
P Values	< 0.001	< 0.001	< 0.001
Q3, > 9.83	18.24[16.15,20.64]	19.01[16.76,21.6]	11.57[10.11,13.27]
P Values	< 0.001	< 0.001	< 0.001
P for Trend	< 0.001	< 0.001	< 0.001

Data are presented as common odds ratios [95% confidence intervals]. Model 1 was unadjusted; Model 2 adjusted for gender, ethnicity, income, education, housing instability, regular health-care access, and food security; Model 3 adjusted for Model 2 + sleep problem, physical activity, health eating index score, smoking, depression and anti-hypertension drug. P for trend was calculated using the median of ePWV in each tertiles. ePWV, estimated pulse wave velocity

### Association between ePWV and both all-cause and cardiovascular mortality in participants with different CKM stages

**Fig. 1:**
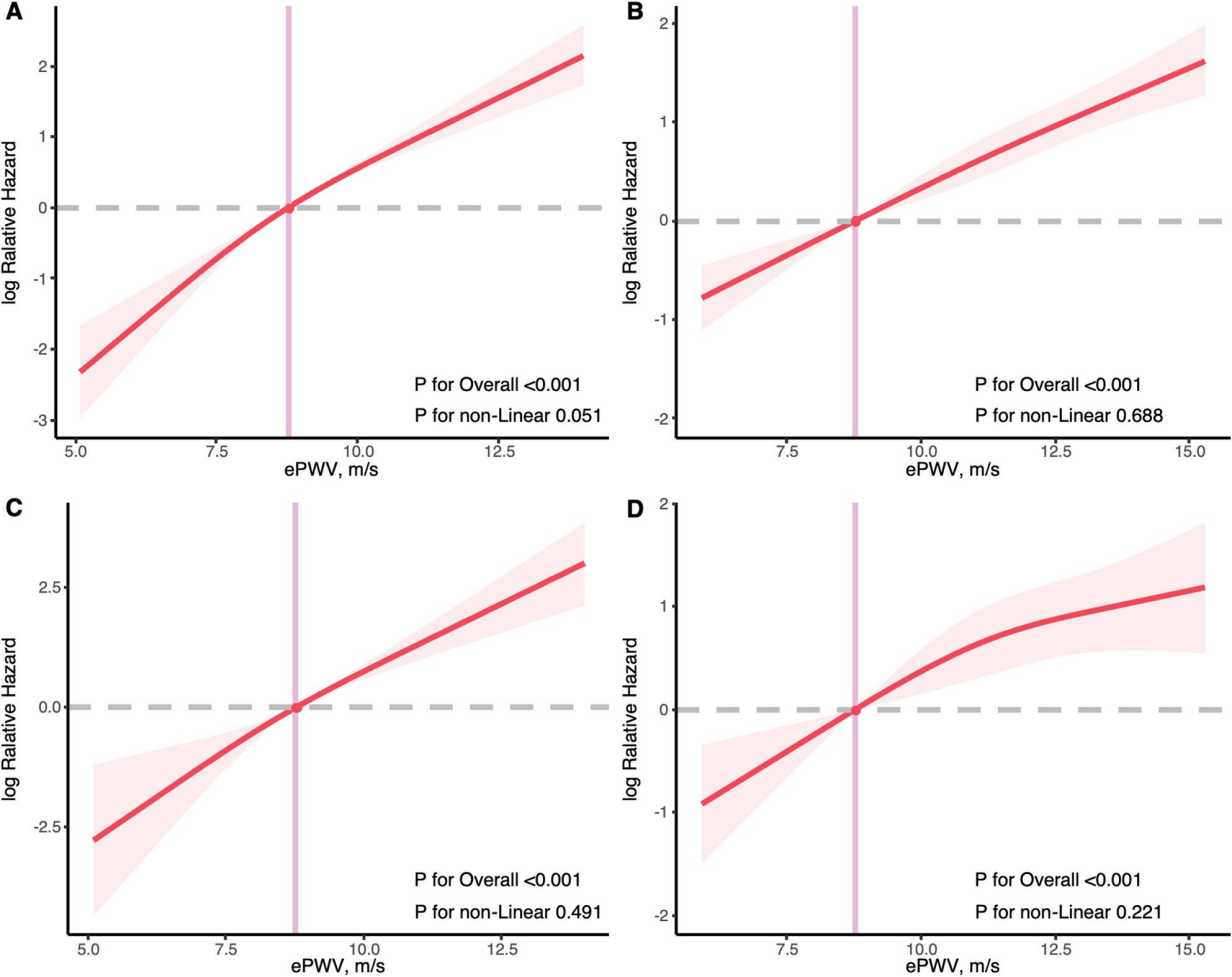
Adjusted relative hazard for the association between ePWV and all-cause and cardiovascular mortality in individuals with early and advanced CKM. **A**, association between ePWV and all-cause mortality in individuals with early CKM; **B**, association between ePWV and all-cause mortality in individuals with advanced CKM; **C**, association between ePWV and cardiovascular mortality in individuals with early CKM; **D**, association between ePWV and cardiovascular mortality in individuals with advanced CKM. Data were fitted using Cox proportional hazard models with RCS with 3 knots (the 10th, 50th, and 90th percentiles) for estimated pulse wave velocity, adjusted for potential covariates. Reference is the 50th percentile of estimated pulse wave velocity (8.77 m/s). CKM, cardiovascular-kidney-metabolic syndrome; ePWV, estimated pulse wave velocity; RCS, restricted cubic spline.

During the median follow-up duration of 81 months, 1000 participants experienced all-cause mortality and among them 298 experienced cardiovascular mortality. Table 2 demonstrates a significant association between ePWV and all-cause mortality among participants with both early CKM and advanced CKM. The hazard ratios (HR) for each 1 m/s increase in ePWV were 1.60 [95% CI, 1.51–1.69] for participants with early CKM and 1.29 [95% CI, 1.23–1.35] for those with advanced CKM. When comparing the participants with the lowest ePWV, the HRs for those with highest ePWV were 9.24 [95% CI, 6.78–12.59] for individuals with early CKM and 3.14 [95% CI, 2.48–3.99] for those with advanced CKM. RCS indicated no significant non-linear association (both P for non-linearity > 0.05, Fig. 1A and B). Supplementary Figure S5 illustrates that this association persists across various subgroups, with significant interactions observed between ePWV and both age and hypertension among participants with early CKM. Additionally, a significant interaction was found between ePWV and waist circumference in individuals with advanced CKM. The complete-case analysis yielded similar results (Supplementary Table S4).

**Table 3:** Associations between estimated pulse wave velocity and both all-cause and cardiovascular mortality in individuals with early and advanced CKM

ePWV	Early CKM			Advanced CKM		
	Model1	Model2	Model3	Model1	Model2	Model3
**All-cause mortality**						
**Continuous**						
Per 1 m/s increase	1.53 [1.45, 1.60]	1.53 [1.45, 1.61]	1.60 [1.51, 1.69]	1.27 [1.22, 1.33]	1.27 [1.21, 1.33]	1.29 [1.23, 1.35]
P Values	< 0.001	< 0.001	< 0.001	< 0.001	< 0.001	< 0.001
**Tertiles of ePWV**,** m/s**						
Q1, < 7.79	Ref	Ref	Ref	Ref	Ref	Ref
P Values	-	-	-	-	-	-
Q2, 7.79 ~ 9.83	2.62 [1.93, 3.55]	2.82 [2.07, 3.84]	2.91 [2.13, 3.98]	2.00 [1.60, 2.50]	1.95 [1.55, 2.45]	2.06 [1.62, 2.60]
P Values	< 0.001	< 0.001	< 0.001	< 0.001	< 0.001	< 0.001
Q3, > 9.83	7.84 [5.89, 10.42]	7.93 [5.90, 10.66]	9.24 [6.78, 12.59]	3.08 [2.48, 3.81]	2.91 [2.32, 3.64]	3.14 [2.48, 3.99]
P Values	< 0.001	< 0.001	< 0.001	< 0.001	< 0.001	< 0.001
P for Trend	< 0.001	< 0.001	< 0.001	< 0.001	< 0.001	< 0.001
**Cardiovascular mortality**						
**Continuous**						
Per 1 m/s increase	1.69 [1.52, 1.87]	1.74 [1.55, 1.96]	1.84 [1.63, 2.08]	1.26 [1.17, 1.35]	1.23 [1.14, 1.33]	1.23 [1.13, 1.34]
P Values	< 0.001	< 0.001	< 0.001	< 0.001	< 0.001	< 0.001
**Tertiles of ePWV**,** m/s**						
Q1, < 7.79	Ref	Ref	Ref	Ref	Ref	Ref
P Values	-	-	-	-	-	-
Q2, 7.79 ~ 9.83	2.33 [1.15, 4.74]	2.60 [1.27, 5.33]	2.67 [1.30, 5.48]	2.79 [0.98, 7.96]	2.74 [0.96, 7.82]	2.90 [1.01, 8.30]
P Values	0.019	0.009	0.007	0.055	0.06	0.047
Q3, > 9.83	10.53 [5.61, 19.75]	11.57 [6.03, 22.22]	13.76[7.00,27.06]	5.83 [2.16, 15.71]	5.13 [1.89, 13.92]	5.33 [1.95, 14.61]
P Values	< 0.001	< 0.001	< 0.001	< 0.001	0.001	0.001
P for Trend	< 0.001	< 0.001	< 0.001	< 0.001	< 0.001	< 0.001

Data are presented as hazard ratios [95% confidence intervals]. Model 1 was unadjusted; Model 2 adjusted for gender, ethnicity, income, education, housing instability, regular health-care access, and food security; Model 3 adjusted for Model 2 + sleep problem, physical activity, health eating index score, smoking and depression. P for trend was calculated using the median of ePWV in each tertiles. CKM, cardiovascular-kidney-metabolic syndrome; ePWV, estimated pulse wave velocity

**Fig. 2:**
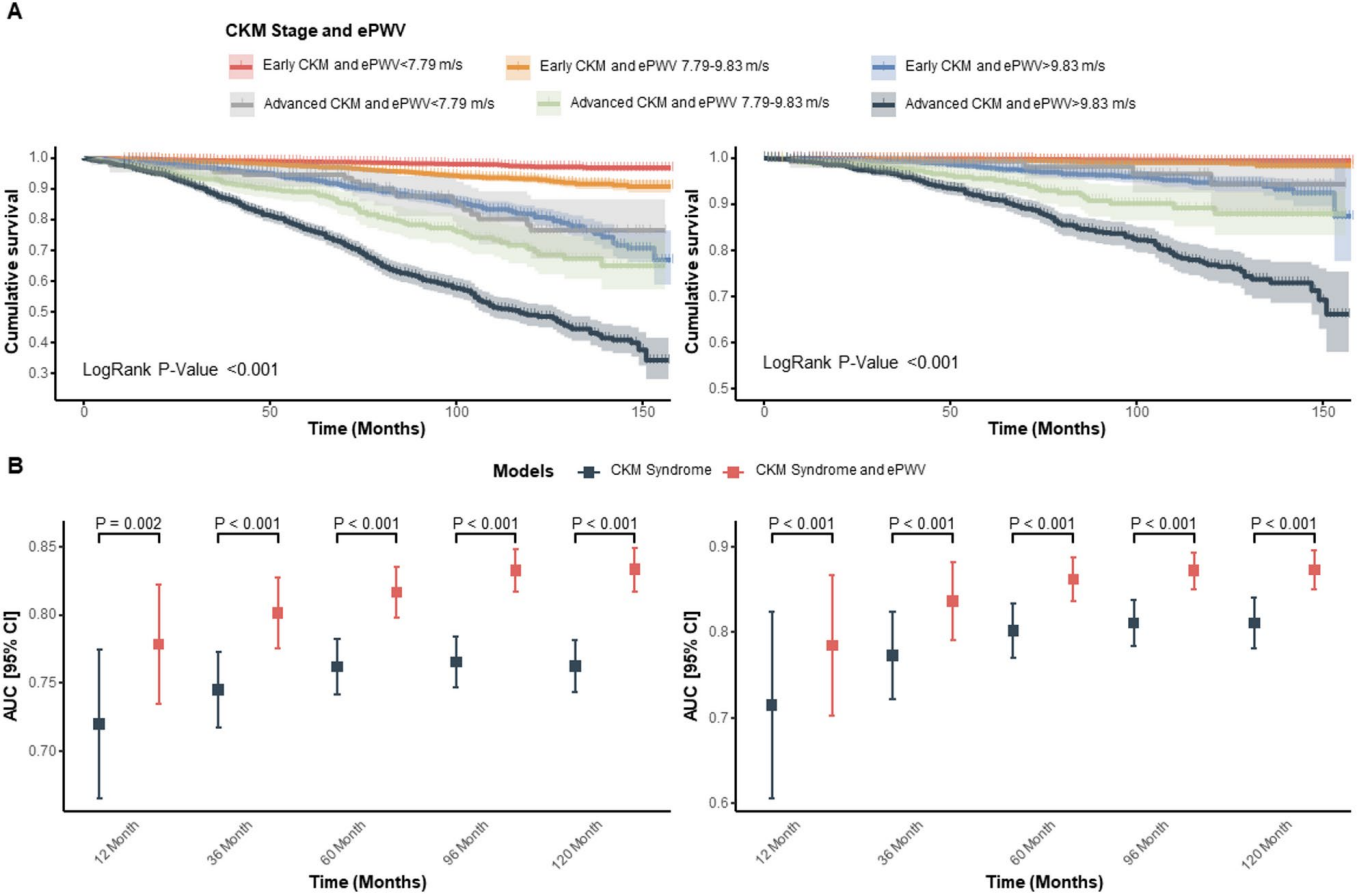
Kaplan–Meier curves and time-dependent AUC. **A**, Kaplan–Meier curves for the survival probability of the all-cause mortality (left) and cardiovascular mortality (right) in the groups with different joint effect of CKM stage and ePWV. **B**, comparison of the predictive value of CKM syndrome combined with ePWV versus CKM syndrome alone for all-cause mortality (left) and cardiovascular mortality (right) across different follow-up periods. AUC, area under curves; CKM, cardiovascular-kidney-metabolic syndrome; ePWV, estimated pulse wave velocity.

The HR for each 1 m/s increase in ePWV associated with cardiovascular mortality was 1.84 [95% CI, 1.63–2.08] for participants with early CKM and 1.23 [95% CI, 1.13–1.34] for those with advanced CKM. For individuals with the highest ePWV, the HRs were 13.76 [95% CI, 7.00-27.06] for participants with early CKM and 5.33 [95% CI, 1.95–14.61] for those with advanced CKM. RCS indicated no significant non-linear association (both P for non-linearity > 0.05, as shown in Fig. 1C and D). This association persisted across most subgroups. Additionally, similar results were observed in the complete-case analysis and competing risk models (Supplementary Table S5).

### Joint association between CKM stage and ePWV with all-cause and cardiovascular mortality

The Kaplan-Meier curves (Fig. 2A) demonstrate that participants with advanced CKM and the highest levels of ePWV experienced the lowest survival rates. Furthermore, participants with early-stage CKM and the highest ePWV, along with those with advanced CKM and low to moderate ePWV, presented a significantly higher risk of mortality when compared to individuals with early-stage CKM and low to moderate ePWV. This observation implies a potential interaction between the CKM stage and ePWV.

**Fig. 3:**
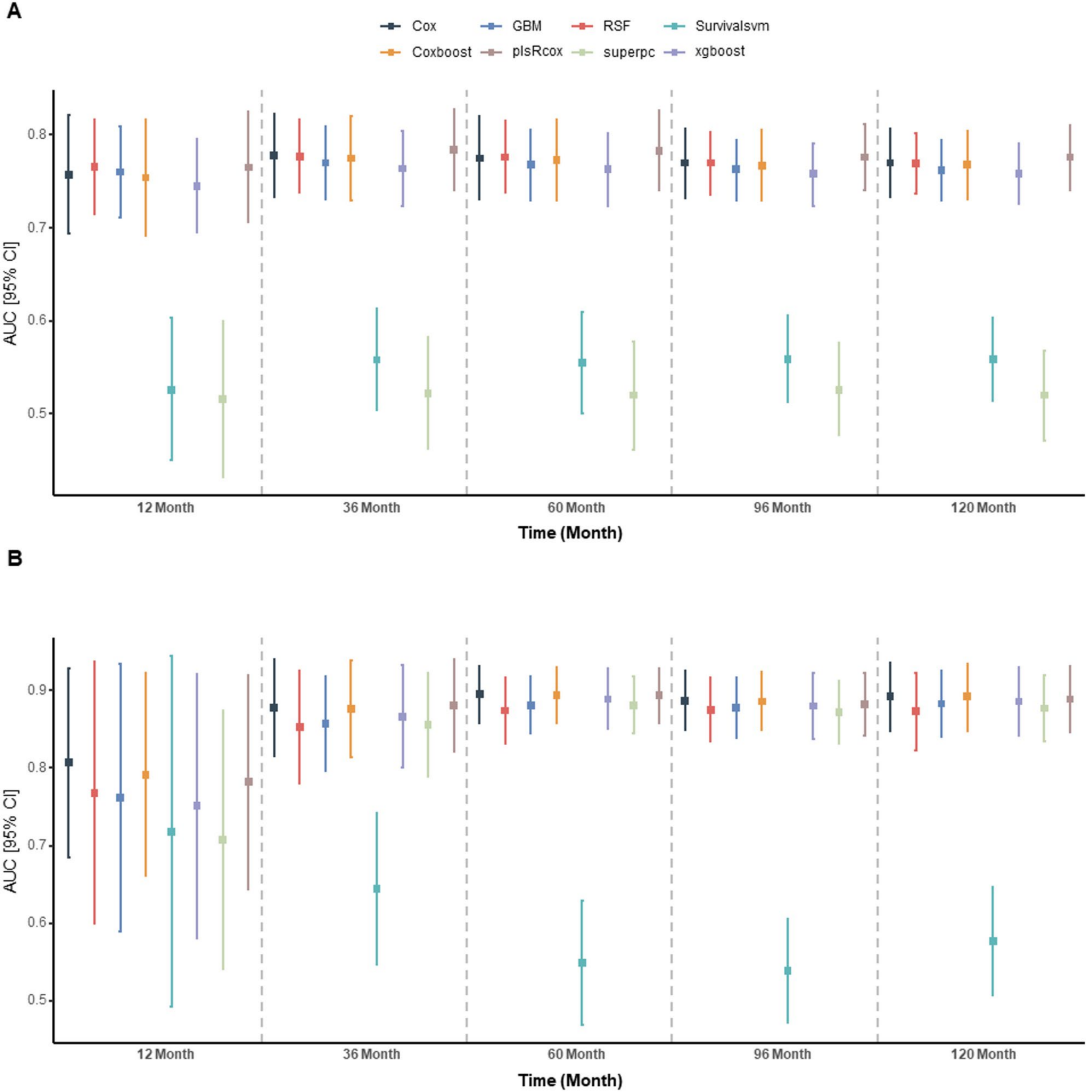
Time-Dependent AUC for predicting all-cause mortality (**A**) and cardiovascular mortality (**B**) using variables defining CKM syndrome and ePWV in test part. CKM, cardiovascular-kidney-metabolic syndrome; ePWV, estimated pulse wave velocity; AUC, area under curves; RSF, Random Survival Forest; GBM, Gradient Boosting Machine; Coxboost, Cox model boosting; Survivalsvm, Survival Support Vector Machine; XGBoost, eXtreme Gradient Boosting; SuperPC, Supervised Principal Components; PLSRcox, Partial Least Squares Regression for Cox models.

Subsequent analyses utilizing Cox proportional hazards models revealed markable interactions between CKM stage and ePWV concerning both all-cause mortality and cardiovascular mortality (P for Interaction < 0.001, Table 3). Specifically, individuals with advanced CKM and the highest ePWV exhibited HR for all-cause mortality and cardiovascular mortality of 22.35 [95% CI, 17.01–29.36] and 42.92 [95% CI, 23.61–78.02], respectively.

**Table 4:** Joint association between CKM stage and ePWV with all-cause and cardiovascular mortality

CKM Stage and ePWV	*n*/*N*	Model1	*P* Value	Model2	*P* Value	Model3	*P* Value	*P*_Interaction_
**All-Cause Mortality**								< 0.001
Early CKM and ePWV < 7.79 m/s	62/3002	Ref	-	Ref	-	Ref	-	
Early CKM and ePWV 7.79–9.83 m/s	122/2717	2.61 [1.92, 3.54]	< 0.001	2.74 [2.01, 3.72]	< 0.001	2.76 [2.03, 3.76]	< 0.001	
Early CKM and ePWV > 9.83 m/s	201/1653	7.80 [5.86, 10.37]	< 0.001	7.59[5.69,10.14]	< 0.001	8.12 [6.05, 10.89]	< 0.001	
Advanced CKM and ePWV < 7.79 m/s	20/138	7.98 [4.82, 13.21]	< 0.001	6.72[4.05,11.14]	< 0.001	6.16 [3.71, 10.24]	< 0.001	
Advanced CKM and ePWV 7.79–9.83 m/s	82/421	13.02 [9.36, 18.12]	< 0.001	11.06[7.92,15.43]	< 0.001	10.58 [7.57, 14.79]	< 0.001	
Advanced CKM and ePWV > 9.83 m/s	513/1485	26.17[20.10,34.09]	< 0.001	21.51[16.42,28.16]	< 0.001	22.35 [17.01,29.36]	< 0.001	
P for Trend		< 0.001	-	< 0.001	-	< 0.001	-	
**Cardiovascular Mortality**								< 0.001
Early CKM and ePWV < 7.79 m/s	12/3002	Ref	-	Ref	-	Ref	-	
Early CKM and ePWV 7.79–9.83 m/s	21/2717	2.33 [1.15, 4.74]	0.019	2.43 [1.20, 4.96]	0.014	2.51 [1.23, 5.12]	0.011	
Early CKM and ePWV > 9.83 m/s	52/1653	10.51[5.61,19.69]	< 0.001	10.36 [5.49, 19.55]	< 0.001	11.37 [6.00, 21.57]	< 0.001	
Advanced CKM and ePWV < 7.79 m/s	4/138	8.28[2.67,25.67]	< 0.001	7.35 [2.36, 22.85]	0.001	7.06 [2.26, 22.04]	0.001	
Advanced CKM and ePWV 7.79–9.83 m/s	28/421	23.11[11.75,45.47]	< 0.001	20.73[10.48,41.02]	< 0.001	21.21[10.69,42.08]	< 0.001	
Advanced CKM and ePWV > 9.83 m/s	181/1485	48.23[26.86,86.60]	< 0.001	40.48[22.35,73.33]	< 0.001	42.92 [23.61,78.02]	< 0.001	
P for Trend		< 0.001	-	< 0.001	-	< 0.001	-	

Data are presented as hazard ratios [95% confidence intervals]. Model 1 was unadjusted; Model 2 adjusted for gender, ethnicity, income, education, housing instability, regular health-care access, and food security; Model 3 adjusted for Model 2 + sleep problem, physical activity, health eating index score, smoking and depression. n/N, death/all individuals. P_interaction_, P values for the interaction effect between ePWV (continuous) and CKM stage (early CKM and advanced CKM). CKM, cardiovascular-kidney-metabolic syndrome; ePWV, estimated pulse wave velocity

Complete-case analysis produced similar findings (Supplementary Table S6). Competing risk models further indicated that, after adjusting for competing events, the cardiovascular mortality risk remained significantly elevated among individuals with both advanced CKM and the highest ePWV (Supplementary Table S7).

### The predictive value of ePWV for mortality

Figure 2B shows that the addition of ePWV significantly improved the predictive performance of CKM syndrome for both all-cause and cardiovascular mortality (all P values < 0.05). Compared with the base model (CKM syndrome alone), the combined model exhibited an NRI ranging from 0.25 to 0.382 for all-cause mortality and from 0.005 to 0.431 for cardiovascular mortality (Supplementary Table S8). 

The performance of the various models on the train sets is presented in Supplementary Table S9 and Supplementary Figure S6. The performance of test sets is presented in Fig. 3 and Supplementary Table S10. The time-dependent AUC indicates that the combination of variables used to define CKM syndrome with ePWV provided a robust prediction of mortality risk in most models (time-dependent AUC > 0.7).

### ePWV on CKM syndrome in national representative population

Supplementary Table S11 indicates a significant association between ePWV and CKM stages within the nationally representative population. Supplementary Table S12 demonstrates a significant association between ePWV and both all-cause mortality and cardiovascular mortality across both early and advanced stages of CKM when analyses are conducted using weighted conditions. The Kaplan–Meier curves (Supplementary Figure S7A) indicate that survival rates under weighted analysis are similar to those observed in the unweighted data. Weighted Cox proportional hazards models identified significant interactions between CKM stage and ePWV for both all-cause and cardiovascular mortality (Supplementary Table S13). Specifically, individuals with advanced CKM and the highest ePWV had markedly elevated risks, with HRs of 18.74 (95% CI, 12.36–28.40) for all-cause mortality and 42.08 (95% CI, 20.25–87.47) for cardiovascular mortality. Supplementary Figure S7B shows that incorporating ePWV significantly improves the predictive performance of CKM syndrome for both all-cause and cardiovascular mortality in the weighted analyses (all P values < 0.05).

## Discussion

Previous studies have shown that ePWV exhibits the same predictive value as cfPWV and can effectively reflect the severity of arterial stiffness. Furthermore, owing to its simplicity and ease of acquisition, ePWV has gained extensive utilization in clinical and research settings [14]. Our study conducted a prospective analysis in 9416 participants, and the baseline data showed that individuals with elevated ePWV predominantly exhibited increased BMI, waist circumference, and triglyceride levels. These parameters are hallmarks of central obesity, which serves as a pivotal diagnostic criterion for metabolic syndrome [35]. A cross-sectional study showed a markable association between higher ePWV levels and the increased prevalence of diabetic kidney disease (DKD) [36]. In the present study, we found that ePWV is significantly associated with CKM stages. ePWV demonstrates a non-linear association with CKM stage. Utilizing two-piecewise linear regression analyses, we identified a critical threshold of 7.28 m/s. Consistent with the present study, previous research has also reported non-linear associations between ePWV and metabolic syndrome, as well as CKD [31, 37]. The ePWV threshold of 7.28 m/s represents a critical inflection point that delineates early vascular and metabolic dysfunction from advanced arterial stiffening. Below this threshold, elevations in ePWV reflect emerging pathophysiological processes such as insulin resistance, chronic inflammation, and endothelial dysfunction that that collectively contribute to cardiovascular, renal, and metabolic disease. Timely control of these risk factors can effectively mitigate the progression of arterial stiffness, making ePWV a more sensitive and impactful predictor of subsequent risk events. Once ePWV surpasses this value, significant arterial stiffness is established, and further increases tend to plateau, resulting in a more stable risk relationship [38]. Additionally, individuals with advanced CKM often have concurrent organ damage and multiple comorbidities, which dilutes the relative contribution of ePWV to risk events. This non-linear association further emphasizes the clinical value of ePWV-based early screening for identifying high-risk populations prone to rapid CKM stage progression.

In a secondary analysis of the Systolic Blood Pressure Intervention Trial (SPRINT) study, ePWV serves as a predictor of cardiovascular outcomes. Additionally, among individuals demonstrating a responsive ePWV following intensive antihypertensive treatment, the risk of mortality is reduced by 42% [13, 39]. Furthermore, ePWV notably improves the predictive capacity for mortality risk in diabetic patients, independent of established cardiovascular risk factors [15]. ePWV is a potential indicator for assessing both in-hospital and one-year mortality rates in patients with CKD and CVD, and is significantly associated with elevated cardiovascular and all-cause mortality rates in CKD patients [40]. From a cohort of patients with essential hypertension without known cardiovascular disease, ePWV predicted all-cause mortality, cardiovascular mortality, and composite cardiovascular end point independently of Systematic Coronary Risk Evaluation (SCORE) and Framingham Risk Score [24, 41]. We found that ePWV strongly correlated with both all-cause and cardiovascular mortality across early-stage and advanced-stage CKM populations. Interaction analysis demonstrated that in early-stage CKM patients, ePWV was more strongly associated with all-cause mortality in younger and hypertensive individuals, whereas in advanced CKM, this association was most pronounced among those with higher waist circumferences.

Although the assessment of CKM encompasses many clinical elements, more indicators still need to be evaluated to improve the predictive value of CKM assessment [1]. Our study confirmed a significant interaction between ePWV and CKM syndrome. Thus, we hypotheses that integrating ePWV into CKM syndrome will enhance the accuracy of predicting mortality. To validate this hypothesis, we established two predictive models: Model 1 based on CKM and Model 2 integrating ePWV and CKM. Comparative analyses between the two models confirmed that the inclusion of ePWV significantly improves predictive accuracy for all-cause and cardiovascular mortality. To address potential non-linear relationships, eight machine learning models were developed to further investigate the predictive performance in both the American population and the Chinese population. The results indicate that, across diverse ethnic populations, the incorporation of ePWV and CKM exhibits robust predictive performance for mortality. Given its non-invasive characteristics and ease of measurement, ePWV may serve as a practical tool for large-scale screening, particularly in resource-limited settings [14].

The strengths of this study were that we first established that ePWV had a significant association with CKM in American community residents. Furthermore, ePWV has a strong association with all-cause and cardiovascular mortality in individuals with CKM syndrome. Incorporating ePWV into CKM assessment frameworks significantly improves the predictive accuracy for both all-cause and cardiovascular mortality. These findings underscore the potential utility of ePWV in enhancing risk stratification and guiding therapeutic interventions. Several limitations of our study should be considered when interpreting the results. First, this approach offers precise ePWV measurements within a large aggregated population, but it does not capture longitudinal data on ePWV progression over time, thereby limiting the ability to establish evidence for tracking dynamic changes or temporal phenomena. Second, because most machine learning algorithms do not natively support sampling weights, we did not evaluate the predictive performance of ePWV and the variables used to define CKM syndrome for mortality outcomes under weighted analyses using machine learning methods. Third, the ABI and Carotid IMT are also commonly used indicators of peripheral vascular abnormalities, each reflecting different aspects of peripheral vascular health. However, the presence of missing data in NHANES limits direct comparisons between these indicators and ePWV in this study. Fourth, we did not correct for multiple comparisons in our exploratory subgroup analyses, these results should be interpreted with caution. Fifth, since the cardiovascular benefit drug did not meet the proportional hazards assumption, it was removed from the Cox model. Although we explored the association of ePWV with mortality in patients with different CKM Syndromes, stratified by the use and non-use of cardiovascular benefit drugs in subgroup analysis, unadjusted confounding factors may still be present. Sixth, the absence of cardiovascular mortality data in CHARLS limits the ability to validate the cross-ethnic predictive performance of ePWV in combination with the variables used to define CKM syndrome for cardiovascular mortality. Finally, this study did not present actionable clinical interventions or solutions.

## Conclusion

Our study establishes ePWV as a robust indictor for CKM severity and mortality risk. The incorporation of ePWV into CKM syndrome risk stratification frameworks could provide clinicians with enhanced prognostic accuracy for detecting high-risk individuals, facilitating earlier implementation of targeted therapeutic strategies. RCTs on ePWV-guided interventions across CKM stages and diverse population is warranted.

## Electronic Supplementary Material

Below is the link to the electronic supplementary material.

Supplementary Material 1

## Data Availability

The data sets used and/or analyzed during the current study are publicly available or from the corresponding author upon reasonable request. All authors verify that all information and materials in the manuscript are original.

## Abbreviations

CKM: Cardiovascular-Kidney-Metabolic
CVD: Cardiovascular disease
CKD: Chronic kidney disease
RAAS: Renin-angiotensin-aldosterone system
cfPWV: Carotid-femoral pulse wave velocity
ePWV: Estimated pulse wave velocity
NHANES: National health and nutrition examination survey
NCHS: National center for health statistics
KDIGO: Disease improving global outcomes
eGFR: Estimated glomerular filtration rate
RSF: Random survival forest
GBM: Gradient boosting machine
NDI: National death index
IQR: Interquartile range
RCS: Restricted cubic splines
AUC: Area under curves
NRI: Net reclassification improvement
IDI: Integrated discrimination improvement
TyG: Triglyceride-glucose
DKD: Diabetic kidney disease
