# Supplementary material for: Prognostic value of estimated pulse wave velocity for all-cause and cardiovascular mortality in individuals with cardiovascular–kidney–metabolic (CKM) syndrome: analyses of NHANES 2007–2018
Source: Diabetol Metab Syndr. 2026 May 14;18:149. doi: 10.1186/s13098-026-02174-4 (PMC13344024; doi:10.1186/s13098-026-02174-4)
Supplement: Supplementary file 1 — Supplementary Material 1 [file 13098_2026_2174_MOESM1_ESM.docx]

**Supplementary Methods**

**Detailed definition of CKM syndrome stages**

Stage 0 CKM included individuals who were not overweight/obese (body mass index <25 kg/m^2^, or <23 kg/m^2^ if Asian ancestry) and without metabolic risk factors (hypertension, hypertriglyceridemia [<135 mg/dL], metabolic syndrome, prediabetes, diabetes) or chronic kidney disease (CKD). Stage 1 CKM included individuals with body mass index ≥25 kg/m^2^ (or ≥23 kg/m^2^ if Asian ancestry), waist circumference ≥88/102 cm in women/men (or if Asian ancestry, ≥80/90 cm in women/men), and/or prediabetes without the presence of other metabolic risk factors or CKD. Stage 2 included individuals with metabolic risk factors (hypertriglyceridemia [≥135 mg/dL], hypertension, metabolic syndrome, diabetes), or CKD. Stage 3 included individuals with very-high-risk CKD or high predicted 10-year CVD risk (≥20% using PREVENT [Predict Risk of cardiovascular disease EVENTs] base model, in which values for age and other risk factors outside the validated PREVENT ranges were imputed as upper and lower limits of these ranges). Stage 4 included individuals with clinical CVD, including coronary heart disease, congestive heart failure, and stroke. ^1, 2^ Hypertension is defined as a previous diagnosis of hypertension, blood pressure ≥130/80 mmHg, or current use of antihypertensive medication.^3^ Prediabetes was defined as HbA1c ≥ 5.7% but less than 6.5%, or a fasting glucose level ≥ 100 mg/dL and< 126mg/dL. Diabetes is defined as a previous diagnosis of diabetes, hemoglobin A1c >6.5%, fasting blood glucose ≥7.0 mmol/L, random blood glucose ≥11.1 mmol/L, two-hour oral glucose tolerance test (OGTT) blood glucose ≥11.1 mmol/L, or current use of antidiabetic medication or insulin.^3^ MetS is defined as meeting three or more of the International Diabetes Federation criteria.^4, 5^

**Detailed definition of other variables**

Age, gender, ethnicity, income level, education, housing instability, regular health-care-access, and food security were required from questionnaires. Age, gender, and ethnicity were self-reported. Income levels were determined using the poverty/income ratio (PIR): PIR ≤ 1.3 was classified as low income, 1.3-3.5 as median income, and >3.5 as high income.^6^ Educational levels was categorized as less than high school or high school and above. ^7^ Housing stability was assessed by asking participants if their current residence was owned, being purchased, rented, or occupied through another arrangement, those still making mortgage payments were classified as homeowners. ^7^ Access to healthcare was evaluated by asking participants if they have a usual place to go when sick or in need of health advice. Respondents who answered “yes” or “there is more than one place” were considered to have a routine place for healthcare, except if the usual place was a hospital emergency room, in which case they were classified as not having a routine place for healthcare. ^7^ Food security status was measured using the 10-item U.S. Food Security Survey Module, with classifications as follows: full food security (no affirmative responses), marginal food security (1-2 affirmative responses), low food security (3-5 affirmative responses), and very low food security (6-10 affirmative responses). ^7^ Sleep problem was defined as sleep less than 7 hours or more than 8 hours per night. ^8^ Regular physical activity was defined as ≥150 min of moderate-intensity activity per week or ≥75 min of vigorous-intensity activity per week or an equivalent combination. Health Eating Index (HEI) was used to access diet quality. ^9^ Depression was defined as Patient Health Questionnaire (PHQ-9) scores of 10 or higher. ^10^ anti-hypertension medicine accordance to 2017 ACC/AHA/AAPA/ABC/ACPM/AGS/APhA/ASH/ASPC/ NMA/PCNA guideline for the prevention, detection, evaluation, and management of high blood pressure in adults. ^11^ Cardiovascular improvement medicine including Angiotensin-Converting Enzyme Inhibitor (ACEI), Angiotensin II Receptor Blocker (ARB), Angiotensin Receptor-Neprilysin Inhibitor (ARNI) and beta blockers.

**China Health and Retirement Longitudinal Study data**

A total of 17,301 participants were initially enrolled in this study, of whom 10,414 who were fasting at the time of blood collection were included. After excluding 3,502 participants due to missing data for the variables defining CKM syndrome or the ePWV variable, a final sample of 6,912 participants was selected as the validation set. The following data were collected for the purposes of this study: demographic data (age, gender); body measurements (systolic blood pressure, diastolic blood pressure, height, weight, and waist circumference); lifestyle data (smoking); data on disease history and medication history (hypertension, hypertension medication, diabetes, and diabetes medication); laboratory test data (Glycated Hemoglobin A1c, fasting blood glucose, total triglycerides, total cholesterol , high-density lipoprotein cholesterol , low-density lipoprotein cholesterol , and serum creatinine). CKD risk was defined according to the Kidney Disease Improving Global Outcomes (KDIGO) guidelines based on eGFR, which was calculated using the Chinese-modified Diet in Renal Disease (C-MDRD) equation. ^1, 12-14^ The 10-year CVD risk was estimated using the Framingham risk score.^14, 15^ Regarding mortality information, field investigators determined the survival status of participants through on-site visits. For deceased participants, investigators gathered relevant information by interviewing family members who lived with the decedent. The follow-up period was defined as the number of years from enrollment to the date of death. Over a 9-year follow-up, a total of 159 participants died.

**Table S1** Hyperparameter search space of machine learning models.

| **Model** | **Hyperparameter Search Space** |
| --- | --- |
| Cox Regression | - |
| Random Survival Forest (RSF) | ntree (500, 1000, 2000);  nodesize (2, 3, 5); mtry (1, 2);  splitrule ("logrank", "extratrees"). |
| Gradient Boosting Machine (GBM) | n.trees (500, 1000, 2000);  interaction.depth (3, 5, 7);  n.minobsinnode (2, 3, 5);  shrinkage (0.001, 0.01, 0.1). |
| XGBoost | eta (0.01, 0.03, 0.1);  max_depth (2, 3, 5);  subsample (0.8, 1);  colsample_bytree (0.8, 1);  gamma (0, 0.5, 1);  nrounds (50, 100, 200). |
| superpc | s0.perc (0.3, 0.5, 0.7);  n.components (1, 3, 5);  min.features (2, 5, 10);  n.threshold (10, 20, 30). |
| CoxBoost | start.penalty (100, 200, 500);  maxstepno (100, 200, 300). |
| plsRcox | nt (1, 3, 5, 10) |
| survivalsvm | gamma.mu (0.01, 0.1, 1);  kernel ("lin_kernel", "radial_kernel");  sgf.sv (3, 5, 10);  sigf (5, 7, 10);  margin (0.01, 0.05, 0.1). |

**Table S2** Baseline characteristics based on estimated pulse wave velocity of complete-case analysis

|  | | ePWV, m/s | | |  |
| --- | --- | --- | --- | --- | --- |
| Characteristic | Overall  N = 5,255^1^ | Q1, <7.55  N = 1,752^1^ | Q2, 7.55-9.46  N = 1,752^1^ | Q3,>9.46  N = 1,751^1^ | p-value^2^ |
| Age, year | 54.69 (13.85) | 40.81 (7.17) | 53.95 (7.55) | 69.30 (7.78) | <0.001 |
| Gender |  |  |  |  | <0.001 |
| Female | 2,120 (40.34%) | 622 (35.50%) | 748 (42.69%) | 750 (42.83%) |  |
| Male | 3,135 (59.66%) | 1,130 (64.50%) | 1,004 (57.31%) | 1,001 (57.17%) |  |
| Ethnicity |  |  |  |  | <0.001 |
| Other Hispanic | 524 (9.97%) | 194 (11.07%) | 186 (10.62%) | 144 (8.22%) |  |
| Mexican American | 716 (13.63%) | 286 (16.32%) | 259 (14.78%) | 171 (9.77%) |  |
| Non-Hispanic Asian | 343 (6.53%) | 116 (6.62%) | 110 (6.28%) | 117 (6.68%) |  |
| Non-Hispanic Black | 980 (18.65%) | 287 (16.38%) | 377 (21.52%) | 316 (18.05%) |  |
| Non-Hispanic White | 2,692 (51.23%) | 869 (49.60%) | 820 (46.80%) | 1,003 (57.28%) |  |
| Income |  |  |  |  | <0.001 |
| Low | 2,011 (38.27%) | 674 (38.47%) | 717 (40.92%) | 620 (35.41%) |  |
| Median | 1,958 (37.26%) | 615 (35.10%) | 592 (33.79%) | 751 (42.89%) |  |
| High | 1,286 (24.47%) | 463 (26.43%) | 443 (25.29%) | 380 (21.70%) |  |
| Education |  |  |  |  | 0.7 |
| Less than high school | 1,022 (19.45%) | 332 (18.95%) | 338 (19.29%) | 352 (20.10%) |  |
| High School graduate or higher | 4,233 (80.55%) | 1,420 (81.05%) | 1,414 (80.71%) | 1,399 (79.90%) |  |
| Housing Instability |  |  |  |  | <0.001 |
| Own home | 1,496 (28.47%) | 653 (37.27%) | 511 (29.17%) | 332 (18.96%) |  |
| Rent home or other arrangement | 3,759 (71.53%) | 1,099 (62.73%) | 1,241 (70.83%) | 1,419 (81.04%) |  |
| Regular health-care access |  |  |  |  | <0.001 |
| At least one regular health-care facility | 726 (13.82%) | 385 (21.97%) | 225 (12.84%) | 116 (6.62%) |  |
| None or emergency room | 4,529 (86.18%) | 1,367 (78.03%) | 1,527 (87.16%) | 1,635 (93.38%) |  |
| Food security |  |  |  |  | <0.001 |
| Full security | 4,445 (84.59%) | 1,441 (82.25%) | 1,443 (82.36%) | 1,561 (89.15%) |  |
| Marginal Security | 361 (6.87%) | 128 (7.31%) | 148 (8.45%) | 85 (4.85%) |  |
| Low Security | 302 (5.75%) | 118 (6.74%) | 112 (6.39%) | 72 (4.11%) |  |
| Very low Security | 147 (2.80%) | 65 (3.71%) | 49 (2.80%) | 33 (1.88%) |  |
| Sleep Problem |  |  |  |  | >0.9 |
| No | 2,784 (52.98%) | 923 (52.68%) | 931 (53.14%) | 930 (53.11%) |  |
| Yes | 2,471 (47.02%) | 829 (47.32%) | 821 (46.86%) | 821 (46.89%) |  |
| Regular physical Activity |  |  |  |  | <0.001 |
| Yes | 4,210 (80.11%) | 1,485 (84.76%) | 1,401 (79.97%) | 1,324 (75.61%) |  |
| No | 1,045 (19.89%) | 267 (15.24%) | 351 (20.03%) | 427 (24.39%) |  |
| HEI Score | 54.98 (13.73) | 52.97 (13.31) | 54.60 (13.60) | 57.38 (13.89) | <0.001 |
| Smoke |  |  |  |  | <0.001 |
| Never | 2,731 (51.97%) | 991 (56.56%) | 882 (50.34%) | 858 (49.00%) |  |
| Former | 1,575 (29.97%) | 354 (20.21%) | 505 (28.82%) | 716 (40.89%) |  |
| Current | 949 (18.06%) | 407 (23.23%) | 365 (20.83%) | 177 (10.11%) |  |
| BMI, kg/m^2^ | 29.10 (6.17) | 28.47 (6.02) | 29.95 (6.50) | 28.89 (5.89) | <0.001 |
| Waist Circumference, cm | 100.71 (15.15) | 97.37 (15.25) | 102.52 (15.30) | 102.24 (14.34) | <0.001 |
| Triglycerides, mg/dL | 104.00 (73.00, 153.00) | 98.00 (67.00, 149.00) | 112.00 (77.00, 162.00) | 104.00 (76.00, 147.00) | <0.001 |
| HDL, mg/dL | 53.95 (16.21) | 52.10 (15.14) | 53.40 (16.35) | 56.36 (16.81) | <0.001 |
| Hypertension |  |  |  |  | <0.001 |
| No | 2,120 (40.34%) | 1,244 (71.00%) | 602 (34.36%) | 274 (15.65%) |  |
| Yes | 3,135 (59.66%) | 508 (29.00%) | 1,150 (65.64%) | 1,477 (84.35%) |  |
| Pre-Diabetes |  |  |  |  | 0.4 |
| No | 2,705 (51.47%) | 923 (52.68%) | 883 (50.40%) | 899 (51.34%) |  |
| Yes | 2,550 (48.53%) | 829 (47.32%) | 869 (49.60%) | 852 (48.66%) |  |
| Diabetes |  |  |  |  | <0.001 |
| No | 4,092 (77.87%) | 1,608 (91.78%) | 1,321 (75.40%) | 1,163 (66.42%) |  |
| Yes | 1,163 (22.13%) | 144 (8.22%) | 431 (24.60%) | 588 (33.58%) |  |
| Chronic Kidney Disease |  |  |  |  | <0.001 |
| Low risk | 4,427 (84.24%) | 1,642 (93.72%) | 1,545 (88.18%) | 1,240 (70.82%) |  |
| Moderate risk | 596 (11.34%) | 91 (5.19%) | 161 (9.19%) | 344 (19.65%) |  |
| High risk | 143 (2.72%) | 16 (0.91%) | 27 (1.54%) | 100 (5.71%) |  |
| Very high risk | 89 (1.69%) | 3 (0.17%) | 19 (1.08%) | 67 (3.83%) |  |
| Metabolic Syndrome |  |  |  |  | <0.001 |
| No | 3,162 (60.17%) | 1,276 (72.83%) | 953 (54.39%) | 933 (53.28%) |  |
| Yes | 2,093 (39.83%) | 476 (27.17%) | 799 (45.61%) | 818 (46.72%) |  |
| Depression |  |  |  |  | 0.074 |
| No | 4,883 (92.92%) | 1,624 (92.69%) | 1,613 (92.07%) | 1,646 (94.00%) |  |
| Yes | 372 (7.08%) | 128 (7.31%) | 139 (7.93%) | 105 (6.00%) |  |
| Anti-hypertension Drug |  |  |  |  | <0.001 |
| No | 3,326 (63.29%) | 1,544 (88.13%) | 1,114 (63.58%) | 668 (38.15%) |  |
| Yes | 1,929 (36.71%) | 208 (11.87%) | 638 (36.42%) | 1,083 (61.85%) |  |
| Medicine Usage |  |  |  |  | <0.001 |
| No | 3,702 (70.45%) | 1,586 (90.53%) | 1,241 (70.83%) | 875 (49.97%) |  |
| Yes | 1,553 (29.55%) | 166 (9.47%) | 511 (29.17%) | 876 (50.03%) |  |
| CKM Stage |  |  |  |  | <0.001 |
| Stage 0 | 274 (5.21%) | 202 (11.53%) | 60 (3.42%) | 12 (0.69%) |  |
| Stage 1 | 936 (17.81%) | 596 (34.02%) | 250 (14.27%) | 90 (5.14%) |  |
| Stage 2 | 3,134 (59.64%) | 894 (51.03%) | 1,261 (71.97%) | 979 (55.91%) |  |
| Stage 3 | 291 (5.54%) | 3 (0.17%) | 13 (0.74%) | 275 (15.71%) |  |
| Stage 4 | 620 (11.80%) | 57 (3.25%) | 168 (9.59%) | 395 (22.56%) |  |

^1^ Median (IQR) or Mean (SD); n (%)

^2^Kruskal-Wallis rank sum test; Pearson's Chi-squared test

ePWV, estimated pulse wave velocity; HEI, health eating index; BMI, body mass index; HDL, high density lipoprotein; CKM, cardiovascular-kidney-metabolic syndrome

**Table S3** Results for the association between ePWV and cardiovascular-kidney-metabolic syndrome stages in complete-case analysis

| ePWV | Model1 | Model 2 | Model 3 |
| --- | --- | --- | --- |
| **Continuous** |  |  |  |
| Per 1 m/s increase | 1.86[1.81,1.93] | 1.91[1.85,1.98] | 1.77[1.71,1.84] |
| P Values | <0.001 | <0.001 | <0.001 |
| **Tertiles of ePWV, m/s** |  |  |  |
| Q1, <7.55 | Ref | Ref | Ref |
| P Values | - | - | - |
| Q2, 7.55 - 9.46 | 3.69[3.21,4.25] | 4[3.47,4.62] | 3.04[2.63,3.53] |
| P Values | <0.001 | <0.001 | <0.001 |
| Q3, >9.46 | 15.9[13.53,18.74] | 17.64[14.91,20.91] | 10.24[8.56,12.27] |
| P Values | <0.001 | <0.001 | <0.001 |
| P for Trend | <0.001 | <0.001 | <0.001 |

Data are presented as common odds ratios [95% confidence intervals]. Model 1 was unadjusted; Model 2 adjusted for gender, ethnicity, income, education, housing instability, regular health-care access, and food security; Model 3 adjusted for Model 2 + sleep problem, physical activity, health eating index score, smoking, depression and anti-hypertension drug. P for trend was calculated using the median of ePWV in each tertiles. ePWV, estimated pulse wave velocity.

**Table S4** Associations between estimated pulse wave velocity and both all-cause and cardiovascular mortality in individuals with early and advanced CKM in complete-case analysis

|  | Early CKM | | | Advanced CKM | | |
| --- | --- | --- | --- | --- | --- | --- |
| ePWV | Model1 | Model2 | Model3 | Model1 | Model2 | Model3 |
| **All-Cause Mortality** | | | | | | |
| **Continuous** |  |  |  |  |  |  |
| Per 1 m/s increase | 1.49 [1.39, 1.60] | 1.51 [1.40, 1.62] | 1.57 [1.45, 1.69] | 1.32 [1.23, 1.43] | 1.32 [1.22, 1.43] | 1.35 [1.24, 1.48] |
| P Values | <0.001 | <0.001 | <0.001 | <0.001 | <0.001 | <0.001 |
| **Tertiles of ePWV, m/s** | | | | | | |
| Q1, <7.55 | Ref | Ref | Ref | Ref | Ref | Ref |
| P Values | - | - | - | - | - | - |
| Q2, 7.55 ~ 9.46 | 2.37 [1.55, 3.62] | 2.49 [1.63, 3.81] | 2.56 [1.67, 3.93] | 1.79 [1.22, 2.63] | 1.65 [1.11, 2.45] | 1.75 [1.16, 2.63] |
| P Values | <0.001 | <0.001 | <0.001 | 0.003 | 0.013 | 0.007 |
| Q3, >9.46 | 6.21 [4.19, 9.20] | 6.86 [4.57, 10.30] | 7.58 [4.97, 11.58] | 3.30 [2.30, 4.73] | 3.02 [2.05, 4.44] | 3.32 [2.20, 5.01] |
| P Values | <0.001 | <0.001 | <0.001 | <0.001 | <0.001 | <0.001 |
| P for Trend | <0.001 | <0.001 | <0.001 | <0.001 | <0.001 | <0.001 |
| **Cardiovascular Mortality** | | | | | | |
| **Continuous** |  |  |  |  |  |  |
| Per 1 m/s increase | 1.63 [1.41, 1.89] | 1.71 [1.46, 2.00] | 1.81 [1.53, 2.15] | 1.28 [1.14, 1.45] | 1.23 [1.08, 1.40] | 1.22 [1.07, 1.40] |
| P Values | <0.001 | <0.001 | <0.001 | <0.001 | 0.002 | 0.004 |
| **Tertiles of ePWV, m/s** | | | | | | |
| Q1, <7.55 | Ref | Ref | Ref | Ref | Ref | Ref |
| P Values | - | - | - | - | - | - |
| Q2, 7.55 ~ 9.46 | 1.90 [0.68, 5.34] | 2.11 [0.75, 5.98] | 2.20 [0.78, 6.23] | 4.77 [0.62, 36.71] | 4.66 [0.60, 36.11] | 4.88 [0.63, 38.05] |
| P Values | 0.224 | 0.158 | 0.138 | 0.133 | 0.141 | 0.13 |
| Q3, >9.46 | 9.46 [3.91, 22.89] | 11.76 [4.70, 29.39] | 13.73 [5.37, 35.11] | 7.91 [1.10, 56.97] | 6.22 [0.85, 45.69] | 6.27 [0.84, 46.82] |
| P Values | <0.001 | <0.001 | <0.001 | 0.04 | 0.072 | 0.073 |
| P for Trend | <0.001 | <0.001 | <0.001 | <0.001 | 0.01 | 0.017 |

Data are presented as hazard ratios [95% confidence intervals]. Model 1 was unadjusted; Model 2 adjusted for gender, ethnicity, income, education, housing instability, regular health-care access, and food security; Model 3 adjusted for Model 2 + sleep problem, physical activity, health eating index score, smoking and depression. P for trend was calculated using the median of ePWV in each tertiles. CKM, cardiovascular-kidney-metabolic syndrome; ePWV, estimated pulse wave velocity.

**Table S5** Associations between estimated pulse wave velocity and both all-cause and cardiovascular mortality in individuals with early and advanced CKM estimated by competing risk models

|  | Early CKM | | | Advanced CKM | | |
| --- | --- | --- | --- | --- | --- | --- |
| ePWV | Model1 | Model2 | Model3 | Model1 | Model2 | Model3 |
| **All-Cause Mortality** | | | | | | |
| **Continuous** |  |  |  |  |  |  |
| Per 1m/s increase | 1.66[1.5,1.83] | 1.71[1.54,1.91] | 1.8[1.61,2.01] | 1.2[1.12,1.28] | 1.17[1.09,1.26] | 1.17[1.08,1.26] |
| P Values | <0.001 | <0.001 | <0.001 | <0.001 | <0.001 | <0.001 |
| **Tertiles of ePWV, m/s** | | | | | | |
| Q1, <7.55 | Ref | Ref | Ref | Ref | Ref | Ref |
| P Values | - | - | - | - | - | - |
| Q2, 7.55 - 9.46 | 2.28[1.12,4.63] | 2.5[1.22,5.14] | 2.58[1.25,5.32] | 2.66[0.94,7.55] | 2.62[0.91,7.49] | 2.78[0.96,8.08] |
| P Values | 0.022 | 0.013 | 0.011 | 0.065 | 0.073 | 0.061 |
| Q3, >9.46 | 9.83[5.24,18.43] | 10.59[5.45,20.58] | 12.53[6.33,24.78] | 5[1.87,13.39] | 4.41[1.62,12.02] | 4.59[1.65,12.75] |
| P Values | <0.001 | <0.001 | <0.001 | 0.001 | 0.004 | 0.004 |
| P for Trend | <0.001 | <0.001 | <0.001 | <0.001 | <0.001 | <0.001 |

Data are presented as hazard ratios [95% confidence intervals]. Model 1 was unadjusted; Model 2 adjusted for gender, ethnicity, income, education, housing instability, regular health-care access, and food security; Model 3 adjusted for Model 2 + sleep problem, physical activity, health eating index score, smoking and depression. P for trend was calculated using the median of ePWV in each tertiles. CKM, cardiovascular-kidney-metabolic syndrome; ePWV, estimated pulse wave velocity.

**Table S6** Joint association between CKM stage and ePWV with all-cause and cardiovascular mortality in complete-case analysis

| CKM Stage and ePWV | n/N | Model1 | P Value | Model2 | P Value | Model3 | P Value | P_Interaction_ |
| --- | --- | --- | --- | --- | --- | --- | --- | --- |
| **All-Cause Mortality** |  |  |  |  |  |  |  | 0.02 |
| Early CKM and ePWV<7.55 m/s | 33/1692 | Ref | - | Ref | - | Ref | - |  |
| Early CKM and ePWV 7.55-9.46 m/s | 62/1571 | 2.38 [1.56, 3.62] | <0.001 | 2.49 [1.63, 3.81] | <0.001 | 2.53 [1.65, 3.86] | <0.001 |  |
| Early CKM and ePWV>9.46 m/s | 102/1081 | 6.22 [4.20, 9.21] | <0.001 | 6.65 [4.46, 9.92] | <0.001 | 6.99 [4.66, 10.50] | <0.001 |  |
| Advanced CKM and ePWV<7.55 m/s | 6/60 | 6.07 [2.54, 14.50] | <0.001 | 5.33 [2.22, 12.78] | <0.001 | 4.78 [1.98, 11.49] | <0.001 |  |
| Advanced CKM and ePWV 7.55-9.46 m/s | 27/181 | 10.94 [6.57, 18.20] | <0.001 | 9.92 [5.94, 16.58] | <0.001 | 9.61 [5.73, 16.12] | <0.001 |  |
| Advanced CKM and ePWV>9.46 m/s | 189/670 | 21.55 [14.87, 31.22] | <0.001 | 18.28 [12.49, 26.77] | <0.001 | 19.01 [12.90, 28.04] | <0.001 |  |
| P for Trend |  | <0.001 | - | <0.001 | - | <0.001 | - |  |
| **Cardiovascular Mortality** |  |  |  |  |  |  |  | 0.002 |
| Early CKM and ePWV<7.55 m/s | 6/1692 | Ref | - | Ref | - | Ref | - |  |
| Early CKM and ePWV 7.55-9.46 m/s | 9/1571 | 1.90 [0.67, 5.33] | 0.225 | 2.03 [0.72, 5.71] | 0.182 | 2.14 [0.76, 6.03] | 0.151 |  |
| Early CKM and ePWV>9.46 m/s | 28/1081 | 9.41 [3.89, 22.74] | <0.001 | 10.14 [4.14, 24.81] | <0.001 | 11.41 [4.63, 28.13] | <0.001 |  |
| Advanced CKM and ePWV<7.55 m/s | 1/60 | 5.61 [0.67, 46.59] | 0.111 | 5.67 [0.68, 47.34] | 0.109 | 5.92 [0.71, 49.65] | 0.101 |  |
| Advanced CKM and ePWV 7.55-9.46 m/s | 12/181 | 26.66 [9.99, 71.11] | <0.001 | 27.69 [10.29, 74.47] | <0.001 | 30.72 [11.36, 83.07] | <0.001 |  |
| Advanced CKM and ePWV>9.46 m/s | 71/670 | 44.17 [19.17, 101.78] | <0.001 | 40.58 [17.26, 95.40] | <0.001 | 44.99[18.99,106.55] | <0.001 |  |
| P for Trend |  | <0.001 | - | <0.001 | - | <0.001 | - |  |

Data are presented as hazard ratios [95% confidence intervals]. Model 1 was unadjusted; Model 2 adjusted for gender, ethnicity, income, education, housing instability, regular health-care access, and food security; Model 3 adjusted for Model 2 + sleep problem, physical activity, health eating index score, smoking and depression. n/N, death/all individuals. P_interaction_, P values for the interaction effect between ePWV (continuous) and CKM stage (early CKM and advanced CKM). CKM, cardiovascular-kidney-metabolic syndrome; ePWV, estimated pulse wave velocity.

**Table S7** Joint association between CKM stage and ePWV with cardiovascular mortality estimated by competing risk models

| CKM Stage and ePWV | n/N | Model1 | P Value | Model2 | P Value | Model3 | P Value |
| --- | --- | --- | --- | --- | --- | --- | --- |
| Early CKM and ePWV<7.79 m/s | 12/3002 | Ref | - | Ref | - | Ref | - |
| Early CKM and ePWV 7.79-9.83 m/s | 21/2717 | 2.27[1.12,4.6] | 0.024 | 2.33[1.14,4.74] | 0.02 | 2.41[1.18,4.91] | 0.015 |
| Early CKM and ePWV>9.83 m/s | 52/1653 | 9.72[5.19,18.21] | <0.001 | 9.5[5.03,17.93] | <0.001 | 10.4[5.5,19.65] | <0.001 |
| Advanced CKM and ePWV<7.79 m/s | 4/138 | 7.69[2.49,23.74] | <0.001 | 6.84[2.19,21.39] | 0.001 | 6.65[2.11,20.99] | 0.001 |
| Advanced CKM and ePWV 7.79-9.83 m/s | 28/421 | 20.5[10.42,40.3] | <0.001 | 18.41[9.22,36.76] | <0.001 | 18.9[9.4,38] | <0.001 |
| Advanced CKM and ePWV>9.83 m/s | 181/1485 | 38.43[21.44,68.9] | <0.001 | 32.36[17.83,58.73] | <0.001 | 34.4[18.92,62.53] | <0.001 |
| P for Trend |  | <0.001 | - | <0.001 | - | <0.001 | - |

Data are presented as hazard ratios [95% confidence intervals]. Model 1 was unadjusted; Model 2 adjusted for gender, ethnicity, income, education, housing instability, regular health-care access, and food security; Model 3 adjusted for Model 2 + sleep problem, physical activity, health eating index score, smoking and depression. n/N, death/all individuals. CKM, cardiovascular-kidney-metabolic syndrome; ePWV, estimated pulse wave velocity.

**Table S8** Incremental predictive value of CKM syndrome combined with ePWV for all-cause and cardiovascular mortality compared to CKM syndrome alone

| Characteristics | 12 Month | 36 Month | 60 Month | 96 Month | 120 Month |
| --- | --- | --- | --- | --- | --- |
| **All-Cause Mortality** | | | | | |
| AUC for CKM Syndrome | 0.72[0.666,0.774] | 0.745[0.717,0.773] | 0.762[0.741,0.783] | 0.765[0.747,0.784] | 0.762[0.743,0.781] |
| AUC for CKM Syndrome and ePWV | 0.778[0.735,0.822] | 0.801[0.776,0.827] | 0.817[0.798,0.836] | 0.833[0.817,0.848] | 0.833[0.817,0.849] |
| P Value* | 0.002 | <0.001 | <0.001 | <0.001 | <0.001 |
| IDI | 0.003[0,0.007] | 0.027[0.018,0.037] | 0.05[0.039,0.063] | 0.08[0.067,0.095] | 0.086[0.068,0.103] |
| P Value | 0.08 | <0.001 | <0.001 | <0.001 | <0.001 |
| NRI | 0.25[0.135,0.379] | 0.321[0.253,0.374] | 0.371[0.324,0.411] | 0.382[0.343,0.412] | 0.376[0.338,0.414] |
| P Value | <0.001 | <0.001 | <0.001 | <0.001 | <0.001 |
| **Cardiovascular Mortality** | | | | | |
| AUC for CKM Syndrome | 0.715[0.607,0.823] | 0.773[0.722,0.824] | 0.802[0.77,0.833] | 0.811[0.784,0.838] | 0.811[0.782,0.84] |
| AUC for CKM Syndrome and ePWV | 0.784[0.703,0.866] | 0.836[0.791,0.882] | 0.862[0.836,0.888] | 0.872[0.851,0.893] | 0.873[0.849,0.896] |
| P Value* | <0.001 | <0.001 | <0.001 | <0.001 | <0.001 |
| IDI | -0.001[-0.003,0.001] | 0.01[0.004,0.015] | 0.025[0.017,0.036] | 0.038[0.027,0.054] | 0.047[0.034,0.069] |
| P Value | 0.219 | <0.001 | <0.001 | <0.001 | <0.001 |
| NRI | 0.005[-0.182,0.213] | 0.336[0.241,0.448] | 0.431[0.362,0.488] | 0.414[0.361,0.481] | 0.426[0.364,0.49] |
| P Value | 0.144 | <0.001 | <0.001 | <0.001 | <0.001 |

*P value for comparing the AUC of CKM syndrome combined with ePWV versus CKM syndrome alone.

AUC, area under curves; CKM, cardiovascular-kidney-metabolic syndrome; ePWV, estimated pulse wave velocity; NRI, net reclassification improvement; IDI, integrated discrimination improvement.

**Table S9** Time-Dependent AUC for predicting all-cause mortality (A) and cardiovascular mortality (B) using variables defining CKM syndrome and ePWV in the train part

| Model | 12 Month | 36 Month | 60 Month | 96 Month | 120 Month | iAUC (95CI) |
| --- | --- | --- | --- | --- | --- | --- |
| All-Cause Mortality | | | | | | |
| Cox | 0.828(0.791-0.865) | 0.829(0.806-0.853) | 0.843(0.825-0.860) | 0.852(0.837-0.867) | 0.849(0.832-0.865) | 0.841(0.832-0.848) |
| RSF | 0.970(0.964-0.976) | 0.977(0.973-0.980) | 0.983(0.980-0.985) | 0.992(0.990-0.994) | 0.992(0.990-0.995) | 0.983(0.976-0.989) |
| GBM | 0.868(0.832-0.903) | 0.860(0.837-0.883) | 0.866(0.849-0.882) | 0.877(0.863-0.890) | 0.872(0.858-0.887) | 0.868(0.863-0.873) |
| Coxboost | 0.827(0.790-0.865) | 0.829(0.805-0.853) | 0.843(0.825-0.860) | 0.852(0.837-0.867) | 0.849(0.833-0.865) | 0.841(0.832-0.849) |
| Survivalsvm | 0.596(0.533-0.659) | 0.555(0.520-0.589) | 0.529(0.503-0.556) | 0.532(0.510-0.555) | 0.524(0.501-0.547) | 0.544(0.530-0.560) |
| xgboost | 0.851(0.816-0.885) | 0.850(0.827-0.872) | 0.858(0.842-0.874) | 0.872(0.859-0.885) | 0.870(0.856-0.884) | 0.861(0.852-0.868) |
| superpc | 0.505(0.441-0.569) | 0.540(0.507-0.573) | 0.547(0.522-0.573) | 0.551(0.529-0.574) | 0.560(0.537-0.582) | 0.543(0.532-0.552) |
| plsRcox | 0.821(0.782-0.859) | 0.829(0.805-0.853) | 0.838(0.821-0.856) | 0.849(0.835-0.864) | 0.848(0.832-0.864) | 0.838(0.829-0.846) |
| Cardiovascular Mortality | | | | | | |
| Cox | 0.878(0.825-0.932) | 0.871(0.830-0.913) | 0.884(0.860-0.908) | 0.880(0.854-0.907) | 0.879(0.851-0.906) | 0.879(0.875-0.882) |
| RSF | 0.994(0.992-0.997) | 0.995(0.994-0.997) | 0.997(0.996-0.999) | 0.998(0.998-0.999) | 0.999(0.998-1.000) | 0.997(0.996-0.998) |
| GBM | 0.936(0.884-0.988) | 0.950(0.921-0.979) | 0.958(0.941-0.975) | 0.951(0.935-0.967) | 0.953(0.935-0.970) | 0.951(0.944-0.955) |
| Coxboost | 0.875(0.821-0.928) | 0.870(0.828-0.912) | 0.882(0.858-0.907) | 0.882(0.856-0.907) | 0.880(0.853-0.907) | 0.878(0.874-0.882) |
| Survivalsvm | 0.703(0.605-0.801) | 0.611(0.529-0.694) | 0.545(0.480-0.609) | 0.606(0.557-0.654) | 0.581(0.533-0.629) | 0.601(0.567-0.642) |
| xgboost | 0.893(0.835-0.952) | 0.906(0.874-0.938) | 0.913(0.893-0.933) | 0.916(0.898-0.935) | 0.919(0.900-0.939) | 0.911(0.905-0.916) |
| superpc | 0.848(0.787-0.908) | 0.855(0.809-0.900) | 0.869(0.840-0.897) | 0.871(0.846-0.897) | 0.869(0.841-0.897) | 0.864(0.857-0.870) |
| plsRcox | 0.870(0.815-0.924) | 0.867(0.824-0.911) | 0.876(0.849-0.903) | 0.880(0.855-0.906) | 0.879(0.852-0.905) | 0.875(0.870-0.879) |

CKM, cardiovascular-kidney-metabolic syndrome; ePWV, estimated pulse wave velocity; AUC, area under curves; RSF, Random Survival Forest; GBM, Gradient Boosting Machine; Coxboost, Cox model boosting; Survivalsvm, Survival Support Vector Machine; XGBoost, eXtreme Gradient Boosting; SuperPC, Supervised Principal Components; PLSRcox, Partial Least Squares Regression for Cox models.

**Table S10** Time-Dependent AUC for predicting all-cause mortality (A) and cardiovascular mortality (B) using variables defining CKM syndrome and ePWV in the test part

| Models | 12 Month | 24 Month | 60 Month | 96 Month | 108 Month | iAUC (95CI) |
| --- | --- | --- | --- | --- | --- | --- |
| All-Cause Mortality | | | | | | |
| Cox | 0.757(0.694-0.821) | 0.778(0.733-0.823) | 0.775(0.731-0.820) | 0.770(0.732-0.807) | 0.770(0.733-0.807) | 0.772(0.764-0.775) |
| RSF | 0.766(0.715-0.817) | 0.777(0.738-0.817) | 0.776(0.738-0.815) | 0.770(0.736-0.803) | 0.769(0.737-0.802) | 0.773(0.768-0.776) |
| GBM | 0.760(0.711-0.809) | 0.770(0.731-0.809) | 0.768(0.730-0.806) | 0.763(0.730-0.795) | 0.762(0.730-0.794) | 0.766(0.761-0.768) |
| Coxboost | 0.754(0.691-0.817) | 0.775(0.730-0.820) | 0.773(0.729-0.817) | 0.767(0.730-0.805) | 0.768(0.731-0.804) | 0.770(0.762-0.772) |
| Survivalsvm | 0.526(0.450-0.603) | 0.558(0.504-0.613) | 0.555(0.501-0.609) | 0.559(0.513-0.606) | 0.559(0.514-0.604) | 0.554(0.543-0.559) |
| xgboost | 0.745(0.695-0.796) | 0.764(0.724-0.804) | 0.763(0.724-0.802) | 0.758(0.724-0.791) | 0.758(0.726-0.791) | 0.760(0.752-0.762) |
| superpc | 0.516(0.432-0.600) | 0.522(0.462-0.582) | 0.520(0.461-0.578) | 0.526(0.477-0.576) | 0.520(0.471-0.568) | 0.521(0.519-0.524) |
| plsRcox | 0.765(0.706-0.825) | 0.784(0.740-0.828) | 0.783(0.740-0.826) | 0.776(0.740-0.812) | 0.776(0.740-0.811) | 0.779(0.771-0.781) |
| Cardiovascular Mortality | | | | | | |
| Cox | 0.807(0.685-0.929) | 0.878(0.815-0.940) | 0.895(0.858-0.931) | 0.887(0.849-0.925) | 0.892(0.848-0.936) | 0.877(0.849-0.891) |
| RSF | 0.768(0.599-0.937) | 0.853(0.780-0.926) | 0.874(0.832-0.916) | 0.875(0.834-0.916) | 0.873(0.823-0.922) | 0.855(0.821-0.874) |
| GBM | 0.762(0.589-0.934) | 0.857(0.796-0.918) | 0.881(0.845-0.918) | 0.878(0.839-0.917) | 0.883(0.841-0.925) | 0.860(0.821-0.881) |
| Coxboost | 0.791(0.661-0.922) | 0.876(0.814-0.938) | 0.894(0.858-0.930) | 0.886(0.849-0.924) | 0.892(0.848-0.935) | 0.873(0.841-0.890) |
| Survivalsvm | 0.718(0.492-0.944) | 0.644(0.546-0.742) | 0.549(0.469-0.629) | 0.538(0.472-0.605) | 0.577(0.507-0.647) | 0.595(0.549-0.639) |
| xgboost | 0.751(0.581-0.921) | 0.866(0.800-0.933) | 0.889(0.851-0.928) | 0.880(0.838-0.922) | 0.886(0.842-0.930) | 0.863(0.820-0.885) |
| superpc | 0.707(0.540-0.874) | 0.856(0.789-0.923) | 0.881(0.844-0.918) | 0.872(0.831-0.912) | 0.877(0.834-0.920) | 0.849(0.789-0.876) |
| plsRcox | 0.782(0.644-0.920) | 0.881(0.821-0.940) | 0.894(0.858-0.929) | 0.882(0.842-0.922) | 0.889(0.846-0.931) | 0.873(0.838-0.890) |

CKM, cardiovascular-kidney-metabolic syndrome; ePWV, estimated pulse wave velocity; AUC, area under curves; RSF, Random Survival Forest; GBM, Gradient Boosting Machine; Coxboost, Cox model boosting; Survivalsvm, Survival Support Vector Machine; XGBoost, eXtreme Gradient Boosting; SuperPC, Supervised Principal Components; PLSRcox, Partial Least Squares Regression for Cox models.

**Table S11** Results for the association between ePWV and cardiovascular-kidney-metabolic syndrome stages in weighted data.

| Estimated Pulse Wave Velocity | Model1 | Model 2 | Model 3 |
| --- | --- | --- | --- |
| **Continuous** |  |  |  |
| Per 1 m/s increase | 1.86[1.8,6.31] | 1.93[1.87,2] | 1.78[1.71,1.85] |
| P Values | <0.001 | <0.001 | <0.001 |
| **Tertiles of ePWV, m/s** |  |  |  |
| Q1, <7.79 | Ref | Ref | Ref |
| P Values | - | - | - |
| Q2, 7.79 ~ 9.83 | 3.59[3.1,4.16] | 4.17[3.58,4.85] | 3.15[2.72,3.64] |
| P Values | <0.001 | <0.001 | <0.001 |
| Q3, >9.83 | 16.45[13.73,19.7] | 18.89[15.66,22.78] | 11.64[9.43,14.37] |
| P Values | <0.001 | <0.001 | <0.001 |
| P for Trend | <0.001 | <0.001 | <0.001 |

Data are presented as common odds ratios [95% confidence intervals]. Model 1 was unadjusted; Model 2 adjusted for gender, ethnicity, income, education, housing instability, regular health-care access, and food security; Model 3 adjusted for Model 2 + sleep problem, physical activity, health eating index score, smoking, depression and anti-hypertension drug. P for trend was calculated using the median of ePWV in each tertiles. ePWV, estimated pulse wave velocity.

**Table S12** Associations between estimated pulse wave velocity and both all-cause and cardiovascular mortality in individuals with early and advanced CKM in weighted data.

| ePWV | Early CKM | | | Advanced CKM | | |
| --- | --- | --- | --- | --- | --- | --- |
|  | Model1 | Model2 | Model3 | Model1 | Model2 | Model3 |
| **All-Cause Mortality** | | | | | | |
| **Continuous** |  |  |  |  |  |  |
| Per 1 m/s increase | 1.52 [1.42, 1.63] | 1.55 [1.44, 1.67] | 1.61 [1.49, 1.74] | 1.31 [1.24, 1.38] | 1.30 [1.23, 1.37] | 1.33 [1.25, 1.41] |
| P Values | <0.001 | <0.001 | <0.001 | <0.001 | <0.001 | <0.001 |
| **Tertiles of ePWV, m/s** | | | | | | |
| Q1, <7.79 | Ref | Ref | Ref | Ref | Ref | Ref |
| P Values | - | - | - | - | - | - |
| Q2, 7.79 ~ 9.83 | 1.91 [1.22, 2.99] | 2.05 [1.28, 3.26] | 2.09 [1.32, 3.31] | 1.46 [0.87, 2.47] | 1.30 [0.78, 2.15] | 1.33 [0.81, 2.19] |
| P Values | 0.005 | 0.003 | 0.002 | 0.156 | 0.31 | 0.262 |
| Q3, >9.83 | 6.71 [4.54, 9.93] | 7.10 [4.70, 10.72] | 8.14 [5.39, 12.31] | 3.30 [2.00, 5.45] | 2.86 [1.79, 4.55] | 2.99 [1.86, 4.80] |
| P Values | <0.001 | <0.001 | <0.001 | <0.001 | <0.001 | <0.001 |
| P for Trend | <0.001 | <0.001 | <0.001 | <0.001 | <0.001 | <0.001 |
| **Cardiovascular Mortality** | | | | | | |
| **Continuous** |  |  |  |  |  |  |
| Per 1 m/s increase | 1.71 [1.50, 1.94] | 1.80 [1.57, 2.07] | 1.95 [1.73, 2.20] | 1.31 [1.20, 1.43] | 1.30 [1.19, 1.42] | 1.30 [1.17, 1.44] |
| P Values | <0.001 | <0.001 | <0.001 | <0.001 | <0.001 | <0.001 |
| **Tertiles of ePWV, m/s** | | | | | | |
| Q1, <7.79 | Ref | Ref | Ref | Ref | Ref | Ref |
| P Values | - | - | - | - | - | - |
| Q2, 7.79 ~ 9.83 | 2.14 [0.88, 5.24] | 2.28 [0.90, 5.81] | 2.39 [0.99, 5.80] | 2.41 [0.78, 7.49] | 2.24 [0.72, 6.98] | 2.37 [0.75, 7.44] |
| P Values | 0.095 | 0.083 | 0.054 | 0.129 | 0.164 | 0.14 |
| Q3, >9.83 | 10.29[4.29,24.68] | 11.09[4.47,27.50] | 14.63[6.38,33.52] | 5.49 [1.84,16.35] | 4.80 [1.61, 14.29] | 4.84[1.49,15.69] |
| P Values | <0.001 | <0.001 | <0.001 | 0.002 | 0.005 | 0.009 |
| P for Trend | <0.001 | <0.001 | <0.001 | <0.001 | <0.001 | <0.001 |

Data are presented as hazard ratios [95% confidence intervals]. Model 1 was unadjusted; Model 2 adjusted for gender, ethnicity, income, education, housing instability, regular health-care access, and food security; Model 3 adjusted for Model 2 + sleep problem, physical activity, health eating index score, smoking and depression. P for trend was calculated using the median of ePWV in each tertiles. CKM, cardiovascular-kidney-metabolic syndrome; ePWV, estimated pulse wave velocity.

**Table S13** Joint association between CKM stage and ePWV with all-cause and cardiovascular mortality in weighted data.

| CKM Stage and ePWV | n/N | Model1 | P Value | Model2 | P Value | Model3 | P Value | P_Interaction_ |
| --- | --- | --- | --- | --- | --- | --- | --- | --- |
| **All-Cause Mortality** | | | | | | | | 0.004 |
| Early CKM and ePWV<7.79 m/s | 62/3002 | Ref | - | Ref | - | Ref | - |  |
| Early CKM and ePWV 7.79-9.83 m/s | 122/2717 | 1.92 [1.23, 3.00] | 0.004 | 2.00 [1.26,3.16] | 0.003 | 2.01 [1.27, 3.18] | 0.003 |  |
| Early CKM and ePWV>9.83 m/s | 201/1653 | 6.77 [4.60, 9.95] | <0.001 | 6.60 [4.40, 9.89] | <0.001 | 7.01 [4.63, 10.62] | <0.001 |  |
| Advanced CKM and ePWV<7.79 m/s | 20/138 | 6.68 [3.69, 12.08] | <0.001 | 6.06 [3.40, 10.83] | <0.001 | 5.28 [2.92, 9.55] | <0.001 |  |
| Advanced CKM and ePWV 7.79-9.83 m/s | 82/421 | 9.70 [5.98, 15.73] | <0.001 | 7.82 [4.71, 12.97] | <0.001 | 7.40 [4.43, 12.35] | <0.001 |  |
| Advanced CKM and ePWV>9.83 m/s | 513/1485 | 21.82[15.16,31.42] | <0.001 | 18.29 [12.21,27.39] | <0.001 | 18.74[12.36,28.40] | <0.001 |  |
| P for Trend |  | <0.001 | - | <0.001 | - | <0.001 | - |  |
| **Cardiovascular Mortality** | | | | | | | | 0.001 |
| Early CKM and ePWV<7.79 m/s | 12/3002 | Ref | - | Ref | - | Ref | - |  |
| Early CKM and ePWV 7.79-9.83 m/s | 21/2717 | 2.16 [0.89, 5.26] | 0.088 | 2.24 [0.90, 5.54] | 0.081 | 2.30 [0.94, 5.62] | 0.068 |  |
| Early CKM and ePWV>9.83 m/s | 52/1653 | 10.45 [4.35, 25.10] | <0.001 | 10.59 [4.31, 26.02] | <0.001 | 12.06 [4.93, 29.52] | <0.001 |  |
| Advanced CKM and ePWV<7.79 m/s | 4/138 | 8.41 [2.34, 30.25] | 0.001 | 8.19 [2.26, 29.72] | 0.001 | 7.60 [1.97, 29.33] | 0.003 |  |
| Advanced CKM and ePWV 7.79-9.83 m/s | 28/421 | 19.97 [8.31, 48.01] | <0.001 | 17.75 [7.10, 44.35] | <0.001 | 18.33 [7.29, 46.09] | <0.001 |  |
| Advanced CKM and ePWV>9.83 m/s | 181/1485 | 45.29[22.15,92.59] | <0.001 | 39.22[18.76,82.00] | <0.001 | 42.08[20.25,87.47] | <0.001 |  |
| P for Trend |  | <0.001 | - | <0.001 | - | <0.001 | - |  |

Data are presented as hazard ratios [95% confidence intervals]. Model 1 was unadjusted; Model 2 adjusted for gender, ethnicity, income, education, housing instability, regular health-care access, and food security; Model 3 adjusted for Model 2 + sleep problem, physical activity, health eating index score, smoking and depression. n/N, death/all individuals. Pinteraction , P values for the interaction effect between ePWV (continuous) and CKM stage (early CKM and advanced CKM). CKM, cardiovascular-kidney-metabolic syndrome; ePWV, estimated pulse wave velocity.


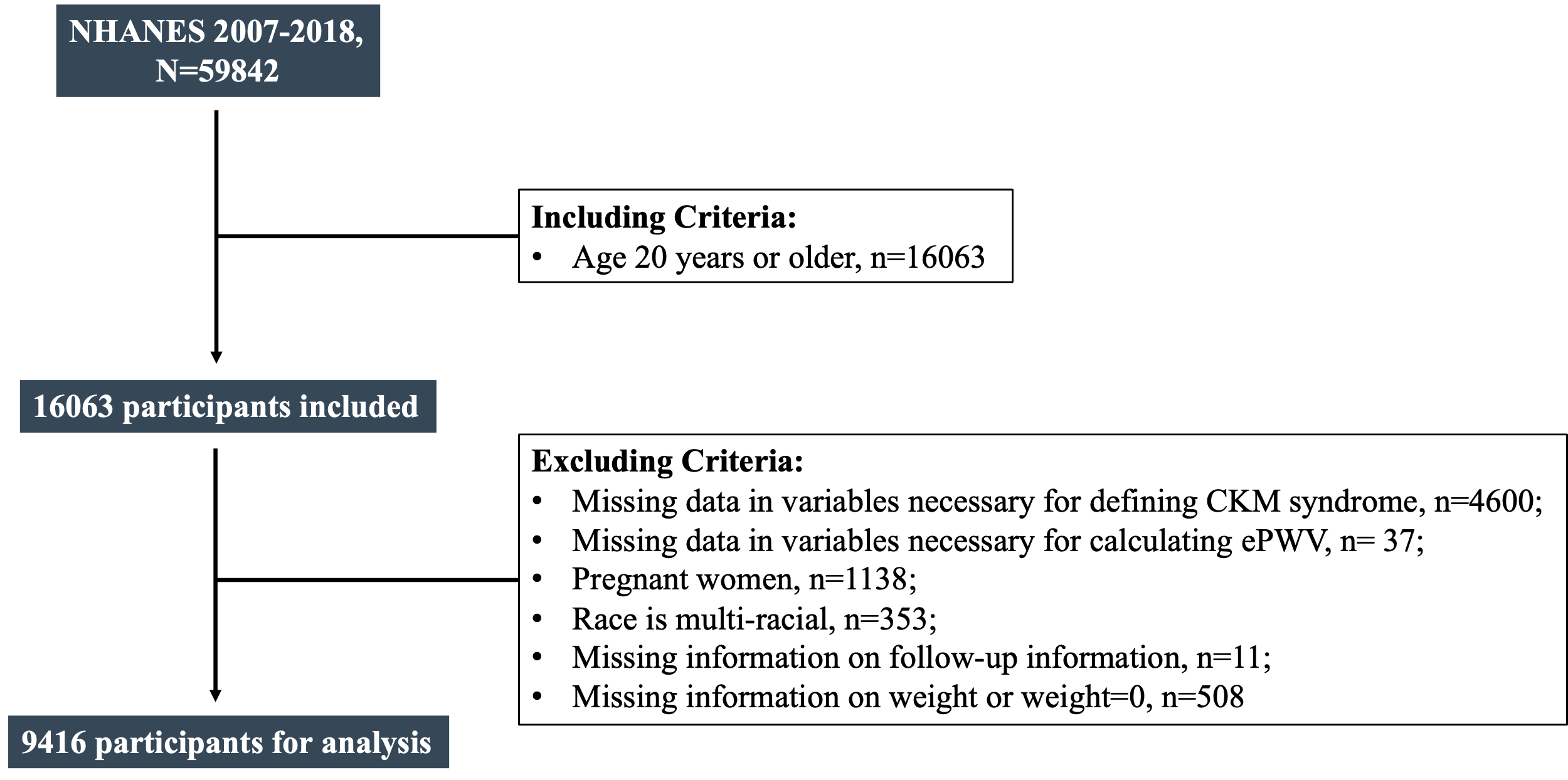


**Figure S1** Flowchart for Participants selection. CKM, cardiovascular-kidney-metabolic syndrome; ePWV, estimated pulse wave velocity.


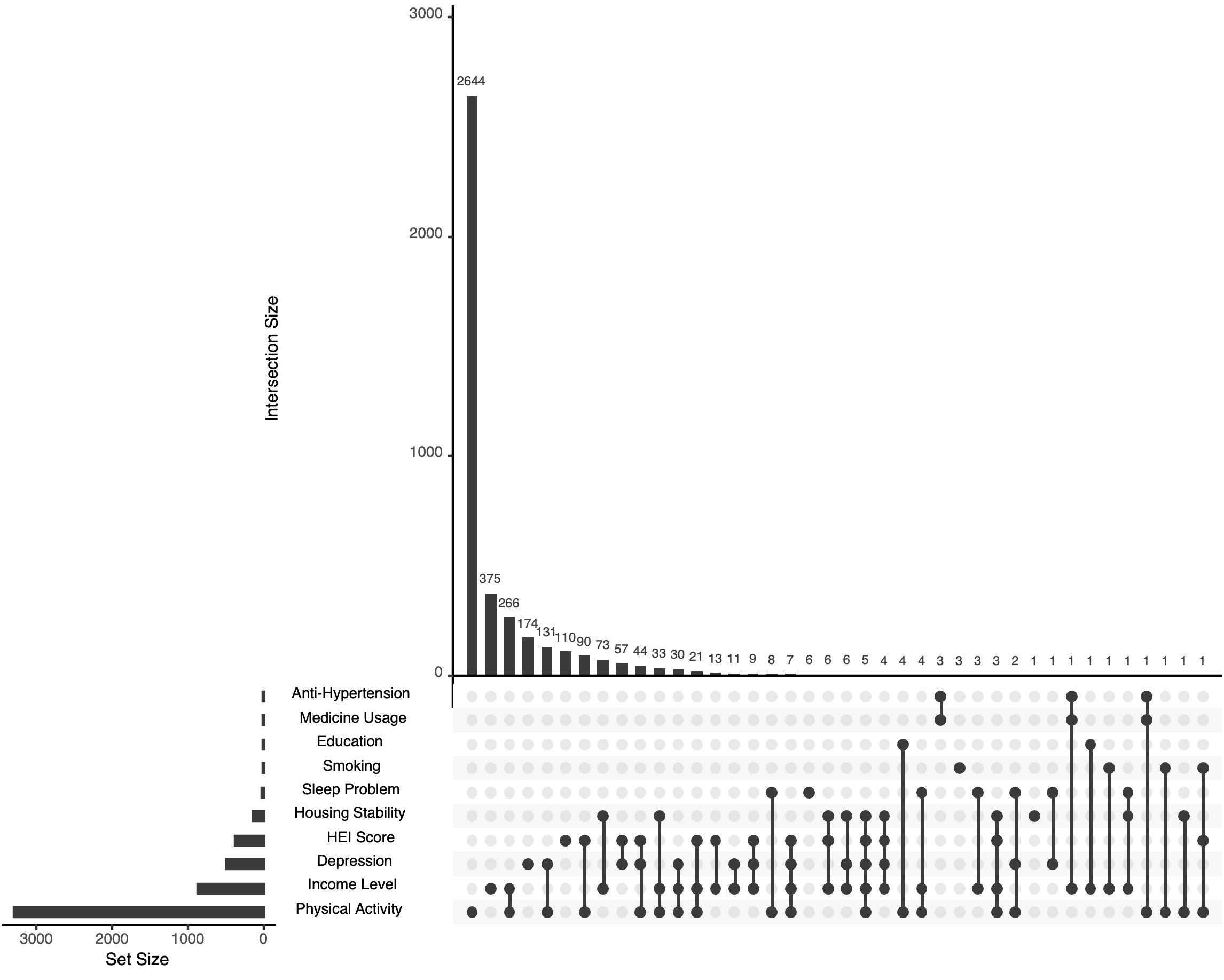


**Figure S2** Patterns of missing data. HEI, health eating index; Medicine usage, Utilization of pharmacological agents with established cardiovascular benefits (ACEI, ARB, ARNI, and β-blockers).


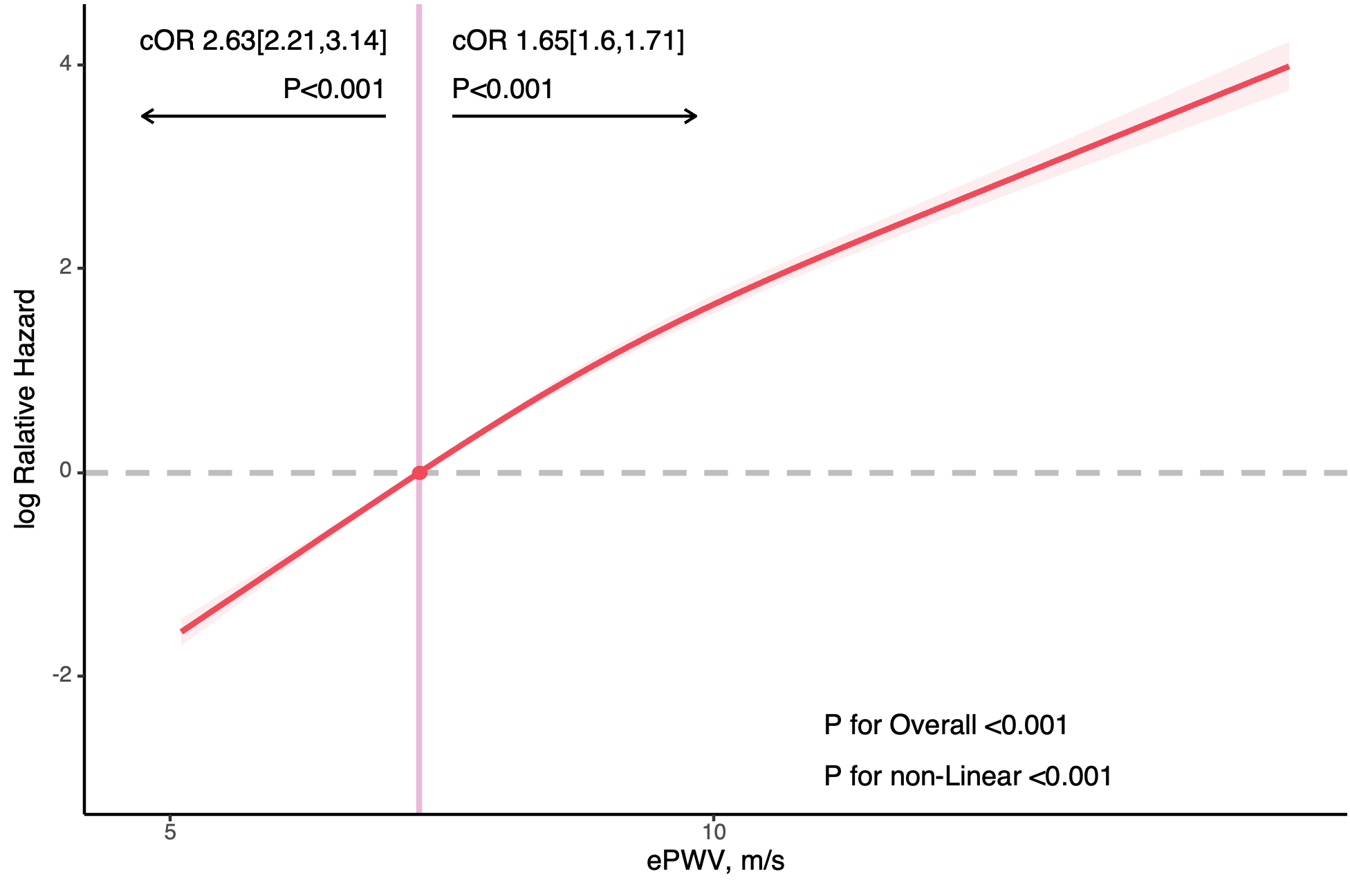


**Figure S3** Adjusted relative hazard for the association between ePWV and CKM stage. Data were fitted using ordinal logical regression model with RCS with 3 knots (the 10th, 50th, and 90th percentiles) for estimated pulse wave velocity, adjusted for potential covariates. Reference is identified by two-piecewise linear regression (7.28 m/s). CKM, cardiovascular-kidney-metabolic syndrome; ePWV, estimated pulse wave velocity; RCS, restricted cubic spline.


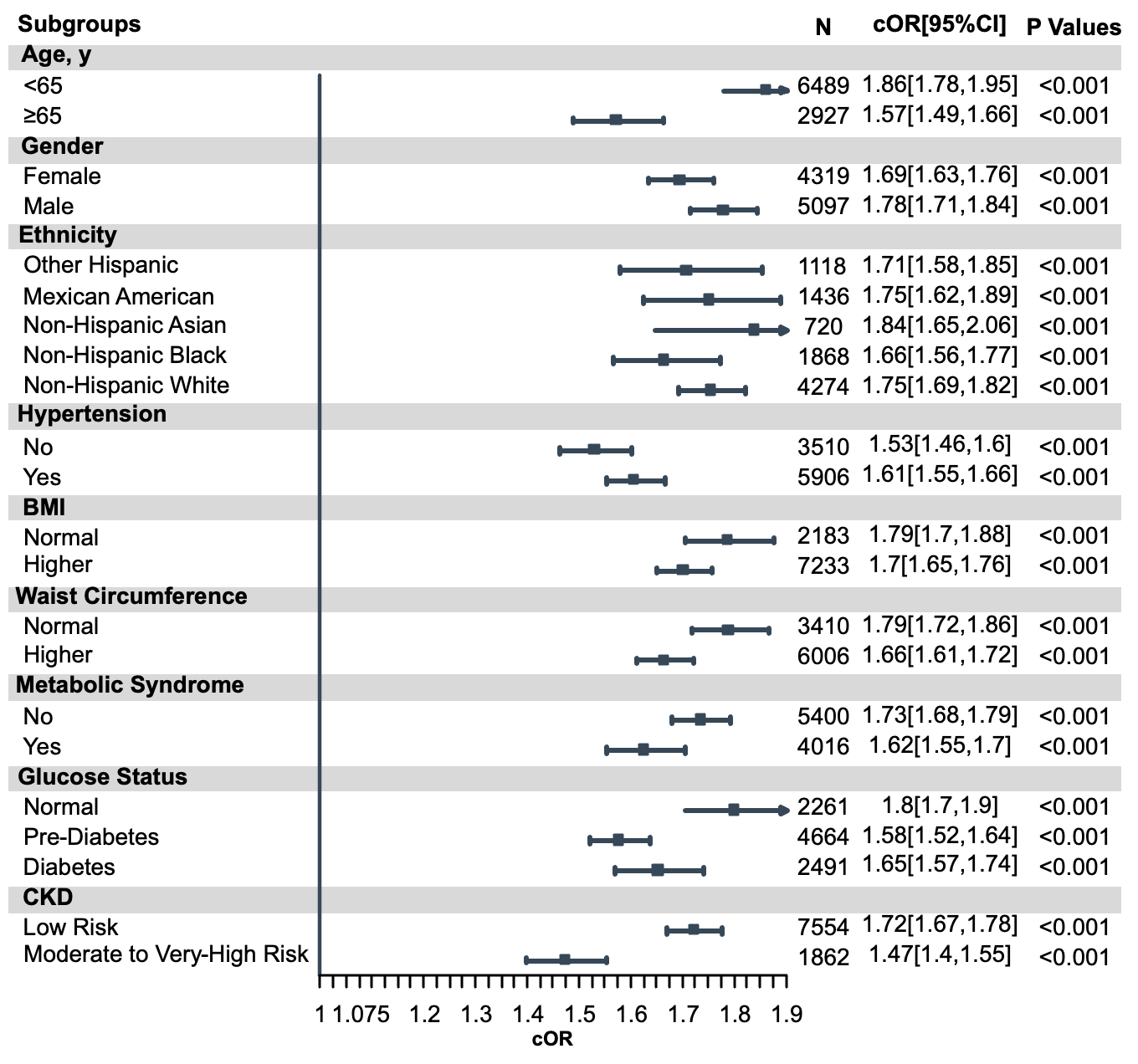


**Figure S4** Association between ePWV and CKM stages in different populations. N, number of persons per subgroup. cOR, common odds ratio; BMI ,body mass index; CKD, chronic kidney disease; CKM, cardiovascular-kidney-metabolic syndrome; ePWV, estimated pulse wave velocity.


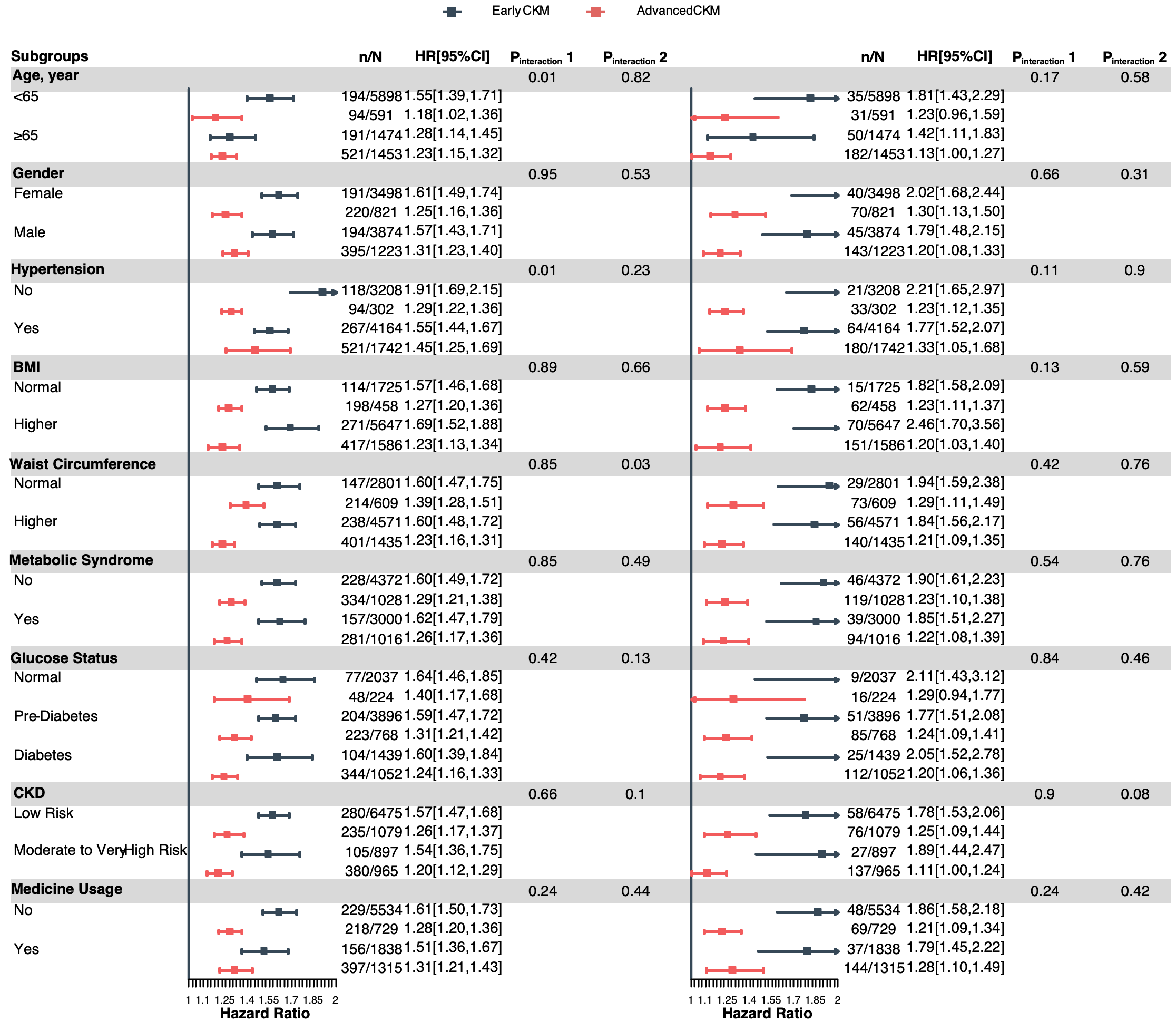


**Figure S5** Associations between estimated pulse wave velocity and both all-cause (left) and cardiovascular mortality(right) in individuals with early and advanced CKM in different populations. n/N, death/all individuals in subgroup; Pinteraction1, the p-value assessing the interaction between ePWV and corresponding subgroup variables in individuals with early CKM；Pinteraction2, the p-value assessing the interaction between ePWV and corresponding subgroup variables in individuals with advanced CKM. For binary variables (age, gender, hypertension, BMI, waist circumference, metabolic syndrome, CKD, and Medicine usage), the interaction term was added directly to the model and its p-value was obtained; for multiple-category variables (glucose status), p-values were calculated by likelihood ratio test, comparing the model with interaction to the model without interaction. BMI is considered normal if it is below 25 kg/m² (or below 23 kg/m² for individuals of Asian ancestry); values above these thresholds are deemed high. Similarly, waist circumference is classified as elevated when it is ≥88 cm in women and ≥102 cm in men (or ≥80 cm in women and ≥90 cm in men for individuals of Asian ancestry), with measurements below these cutoffs considered normal; Medicine usage, Utilization of pharmacological agents with established cardiovascular benefits (ACEI, ARB, ARNI, and β-blockers). BMI, body mass index; CKD, chronic kidney disease; CKM, cardiovascular-kidney-metabolic syndrome; ePWV, estimated pulse wave velocity


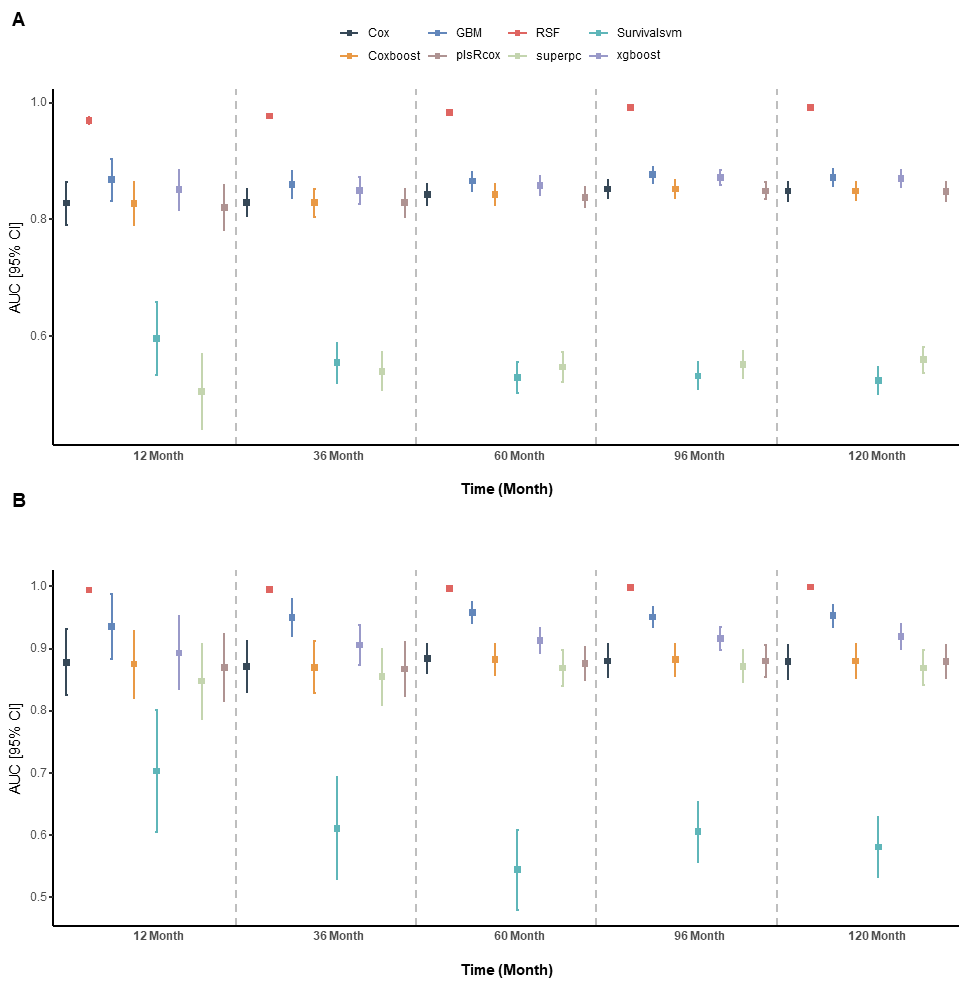


**Figure S6** Time-Dependent AUC for predicting all-cause mortality **(A)** and cardiovascular mortality **(B)** using variables defining CKM syndrome and ePWV in the train part. CKM, cardiovascular-kidney-metabolic syndrome; ePWV, estimated pulse wave velocity; AUC, area under curves; RSF, Random Survival Forest; GBM, Gradient Boosting Machine; Coxboost, Cox model boosting; Survivalsvm, Survival Support Vector Machine; XGBoost, eXtreme Gradient Boosting; SuperPC, Supervised Principal Components; PLSRcox, Partial Least Squares Regression for Cox models.


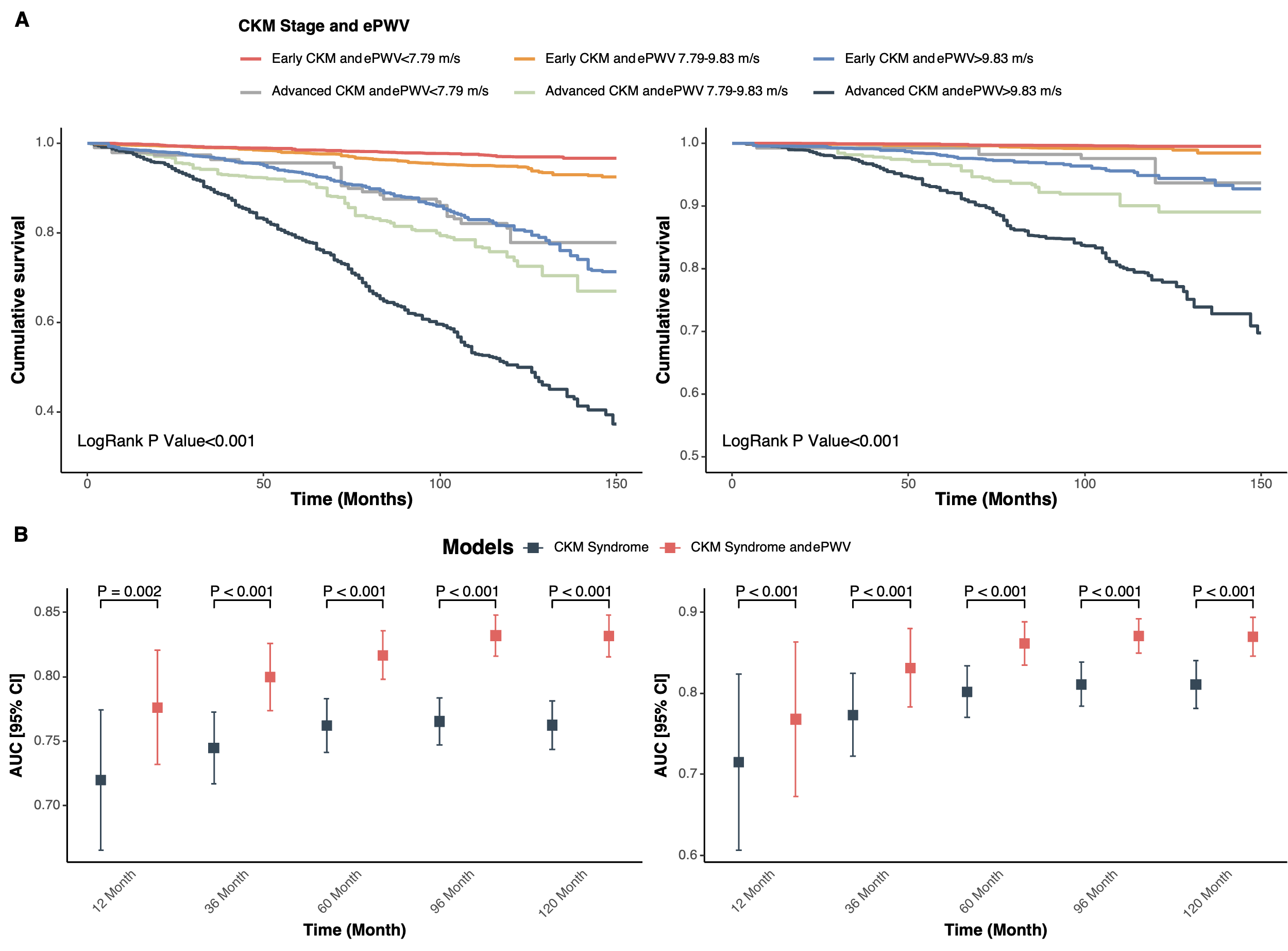


Figure S7 Kaplan–Meier curves and time-dependent AUC in weighted data. A, Kaplan–Meier curves for the survival probability of the all-cause mortality (left) and cardiovascular mortality (right) in the groups with different joint effect of CKM stage and ePWV. B, comparison of the predictive value of CKM syndrome combined with ePWV versus CKM syndrome alone for all-cause mortality (left) and cardiovascular mortality (right) across different follow-up periods. AUC, area under curves; CKM, cardiovascular-kidney-metabolic syndrome; ePWV, estimated pulse wave velocity.

**References**

1. Ndumele CE, Rangaswami J, Chow SL, et al. Cardiovascular-Kidney-Metabolic Health: A Presidential Advisory From the American Heart Association. Circulation. 2023;148(20):1606-35.<https://doi.org/10.1161/cir.0000000000001184>

2. Minhas AMK, Mathew RO, Sperling LS, et al. Prevalence of the Cardiovascular-Kidney-Metabolic Syndrome in the United States. J Am Coll Cardiol. 2024;83(18):1824-6.<https://doi.org/10.1016/j.jacc.2024.03.368>

3. NHANES 1999-2000 Questionnaire Instruments.

4. Alberti KG, Eckel RH, Grundy SM, et al. Harmonizing the metabolic syndrome: a joint interim statement of the International Diabetes Federation Task Force on Epidemiology and Prevention; National Heart, Lung, and Blood Institute; American Heart Association; World Heart Federation; International Atherosclerosis Society; and International Association for the Study of Obesity. Circulation. 2009;120(16):1640-5.<https://doi.org/10.1161/circulationaha.109.192644>

5. Teklu M, Zhou W, Kapoor P, et al. Metabolic syndrome and its factors are associated with noncalcified coronary burden in psoriasis: An observational cohort study. J Am Acad Dermatol. 2021;84(5):1329-38.<https://doi.org/10.1016/j.jaad.2020.12.044>

6. Johnson CL, Paulose-Ram R, Ogden CL, et al. National health and nutrition examination survey: analytic guidelines, 1999-2010. Vital Health Stat 2. 2013(161):1-24.

7. Bundy JD, Mills KT, He H, et al. Social determinants of health and premature death among adults in the USA from 1999 to 2018: a national cohort study. Lancet Public Health. 2023;8(6):e422-e31.<https://doi.org/10.1016/s2468-2667(23)00081-6>

8. Chen LH, Sun SY, Li G, et al. Physical activity and sleep pattern in relation to incident Parkinson's disease: a cohort study. Int J Behav Nutr Phys Act. 2024;21(1):17.<https://doi.org/10.1186/s12966-024-01568-9>

9. Institute. NC. The Healthy Eating Index – Population Ratio Method. Updated December 14, 2021. [Available from: <https://epi.grants.cancer.gov/hei/population-ratio-method.html>.

10. Li W, Ruan W, Peng Y, Lu Z, Wang D. Associations of socioeconomic status and sleep disorder with depression among US adults. J Affect Disord. 2021;295:21-7.<https://doi.org/10.1016/j.jad.2021.08.009>

11. Whelton PK, Carey RM, Aronow WS, et al. 2017 ACC/AHA/AAPA/ABC/ACPM/AGS/APhA/ASH/ASPC/NMA/PCNA Guideline for the Prevention, Detection, Evaluation, and Management of High Blood Pressure in Adults: A Report of the American College of Cardiology/American Heart Association Task Force on Clinical Practice Guidelines. J Am Coll Cardiol. 2018;71(19):e127-e248.<https://doi.org/10.1016/j.jacc.2017.11.006>

12. KDIGO 2021 Clinical Practice Guideline for the Management of Glomerular Diseases. Kidney Int. 2021;100(4s):S1-s276.<https://doi.org/10.1016/j.kint.2021.05.021>

13. Ma YC, Zuo L, Chen JH, et al. Modified glomerular filtration rate estimating equation for Chinese patients with chronic kidney disease. J Am Soc Nephrol. 2006;17(10):2937-44.<https://doi.org/10.1681/asn.2006040368>

14. Li W, Shen C, Kong W, et al. Association between the triglyceride glucose-body mass index and future cardiovascular disease risk in a population with Cardiovascular-Kidney-Metabolic syndrome stage 0-3: a nationwide prospective cohort study. Cardiovasc Diabetol. 2024;23(1):292.<https://doi.org/10.1186/s12933-024-02352-6>

15. D'Agostino RB, Sr., Vasan RS, Pencina MJ, et al. General cardiovascular risk profile for use in primary care: the Framingham Heart Study. Circulation. 2008;117(6):743-53.<https://doi.org/10.1161/circulationaha.107.699579>
